# Distribution and Risk Assessment of Organophosphate Esters in Agricultural Soils and Plants in the Coastal Areas of South China

**DOI:** 10.3390/toxics12040286

**Published:** 2024-04-12

**Authors:** Wangxing Luo, Siyu Yao, Jiahui Huang, Haochuan Wu, Haijun Zhou, Mingjiang Du, Ling Jin, Jianteng Sun

**Affiliations:** 1 School of Environmental Science and Engineering, Guangdong University of Petrochemical Technology, Maoming 525000, China; liubei890118@163.com (W.L.); zhouhaijun_2000@126.com (H.Z.); 15818969010@163.com (M.D.); 2 Iron Man Environmental Technology Co., Ltd., Foshan 528000, China; 3 Department of Civil and Environmental Engineering, The Hong Kong Polytechnic University, Hung Hom, Kowloon 999077, Hong Kong; yaosy19@lzu.edu.cn (S.Y.); ling.jin@polyu.edu.hk (L.J.); 4 School of Housing, Building and Planning, Universiti Sains Malaysia, George Town 11800, Pulau Pinang, Malaysia; wuhaochuan2024@163.com

**Keywords:** organophosphate esters, agriculture soil, plant, ecological risk, human risk assessments

## Abstract

Organophosphate esters (OPEs) are frequently used as flame retardants and plasticizers in various commercial products. While initially considered as substitutes for brominated flame retardants, they have faced restrictions in some countries due to their toxic effects on organisms. We collected 37 soil and crop samples in 20 cities along the coast of South China, and OPEs were detected in all of them. Meanwhile, we studied the contamination and potential human health risks of OPEs. In soil samples, the combined concentrations of eight OPEs varied between 74.7 and 410 ng/g, averaging at 255 ng/g. Meanwhile, in plant samples, the collective concentrations of eight OPEs ranged from 202 to 751 ng/g, with an average concentration of 381 ng/g. TDCIPP, TCPP, TCEP, and ToCP were the main OPE compounds in both plant and soil samples. Within the study area, the contaminants showed different spatial distributions. Notably, higher OPEs were found in coastal agricultural soils in Guangdong Province and crops in the Guangxi Zhuang Autonomous Region. The results of an ecological risk assessment show that the farmland soil along the southern coast of China is at high or medium ecological risk. The average non-carcinogenic risk and the carcinogenic risk of OPEs in soil through ingestion and dermal exposure routes are within acceptable levels. Meanwhile, this study found that the dietary intake of OPEs through food is relatively low, but twice as high as other studies, requiring serious attention. The research findings suggest that the human risk assessment indicates potential adverse effects on human health due to OPEs in the soil–plant system along the coast of South China. This study provides a crucial foundation for managing safety risks in agricultural operations involving OPEs.

## 1. Introduction

Due to their persistence, long-distance atmospheric transport, bioaccumulation, and toxicity, polychlorinated biphenyls (PCBs) and polybrominated diphenyl ethers (PBDEs) have been globally phased out as flame retardants. Consequently, organophosphorus flame retardants (OPFRs) have emerged as alternative options to brominated flame retardants, experiencing widespread adoption globally in recent decades [1]. Organophosphate esters (OPEs) find widespread application as flame retardants and plasticizers in numerous commercial products, including processed plastics, furniture, electronics, construction materials, vehicles, and within the petroleum industry [2,3]. With the development of global urbanization, the consumption of OPEs has steadily increased. As of 2018, the global consumption of OPEs reached 1.05 million tons, representing over 30% of the total flame retardants consumed worldwide [4]. OPEs are primarily mixed with materials through physical addition rather than chemical combination. Consequently, they can readily enter various environmental mediums through processes like volatilization and wear during production, transportation, application, and disposal [5,6,7,8,9,10,11]. These make them more vulnerable to causing pollution in environmental media. Currently, they are also present in water [12,13,14], soil and sediment [15,16], the atmosphere [17,18,19], rain and snow [20], dust [21,22], animals [23,24,25], and plants [26,27,28]. There are three main ways for OPEs to enter the human body: ingestion, respiration, and skin absorption [29,30,31]. They have certain biological toxicity, including reproductive and developmental toxicity, neurotoxicity, endocrine disrupting effects [32,33,34,35], etc. Additionally, chlorinated (Cl) OPEs have been proven to be carcinogenic [36]. Considering the harmful effects of OPEs, their risk to human health has been re-assessed as pollutants, and they have been restricted in some countries.

At present, many researchers have conducted toxicological and epidemiological studies on OPEs. Abdul Qadeer [37] conducted a comparative analysis of PBDEs and OPEs, focusing on their persistence, bioaccumulation, and toxicity (PBT) characteristics across different domains. Striking similarities and overlap between PBDEs and OPEs in terms of their physical and chemical properties, environmental behavior, and global concentrations were unveiled by the research findings. Both of them exhibit potential for biomagnification, bioaccumulation, and food chain transfer, consequently leading to reproductive issues. The irreversible toxic effects, including oxidative stress, liver dysfunction, DNA damage, neurotoxicity, reproductive abnormalities, carcinogenicity, and behavioral alterations, underscore the regrettable nature of substituting OPEs for PBDEs as flame retardants. Sun [38] used Oryzias latipes as an aquatic model to assess the developmental neurotoxicity of OPEs during its early developmental stages. Through embryonic toxicity experiments, it was demonstrated that exposure to OPEs reduces the hatching rate, delays hatching time, increases the occurrence of deformities, and affects the motor behavior and nervous system development of larval fish. Hammel [39] compared the metabolites of OPEs in urine samples from children before and after vaccination and found that exposure to OPEs may be significantly associated with decreased antibody concentrations after vaccination. The metabolites of OPEs, such as DPrHpP and DEHP, were initially discovered to be linked with an elevated risk of Hysteromyoma in premenopausal women [40]. Liu [41] found that long-term exposure to tri-OPEs such as TnBP, TPhP, TMPP, and EHDPP increased the risk of female plasma and female-specific tumors. Specifically, they discovered that EHDPP can lead to breast cancer, while prolonged exposure to the aforementioned chemicals raises the risk of cervical cancer.

Soil plays a critical role as a primary “sink” for various human-created and natural pollutants on our planet, with the majority of the world’s pollutants ultimately ending up in soil [42]. OPEs, being persistent and with high adsorption potential, tend to accumulate in the soil matrix and readily adhere to soil particles because of their hydrophobic nature [43]. When OPEs enter the soil, they produce complex environmental transport and transformations behaviors, potentially affecting deep soil and groundwater through vertical migration, posing non-negligible risks to the ecosystem and human health. Adsorption and transformation are two essential processes that take place in the soil environment, significantly affecting the bioavailability of plants. These processes play a crucial role in determining the fate and behavior of organic pollutants in soil, ultimately influencing their potential impact on plant health and ecosystem dynamics. Wang measured tri-OPEs in 40 soil samples from the wetland soil of the Laizhou Bay of China and found that the concentration of Σ18tri-OPEs ranged from 137 to 386 ng/g dw, with an average concentration of 282 ng/g [44]. You [45] investigated the contamination of tri-OPEs in Tibetan soils and showed that the concentration of Σtri-OPEs ranged from 29.74 to 73.85 ng/g, with a mean value of 50.80 ng/g. The detection rates of TCPP, TPhP, and EHDPP in the nine tri-OPEs were 100%, and that of TDCIPP was the lowest at 6.25%. Liu [46] detected four tri-OPEs in soil samples from a coking plant in Shanxi: TBP, TCIPP, TCPP, and TEP. In an e-waste dismantling park in South China, the study revealed a concentration range of Σtri-OPEs from 288 to 8.03 × 10^4^ ng/g. TPhP was identified as the main tri-OPE in the soil, with a detection frequency of 84.1% and a median concentration of 1.07 × 10^4^ ng/g. The second most common tri-OPE in the soil was TCPP, which was detected with a frequency of 77.6% and a median concentration of 899 ng/g [47]. The above results indicate that tri-OPEs are commonly found in the soil environment in China.

Research on OPEs in crops is still in the early stages compared to research on their presence in soils. Yu [48] conducted a study on the biotransformation behavior and toxicity of OPEs through hydroponic experiments. The findings revealed that OPEs with lower hydrophobicity exhibited greater ease of translocation to the top. In addition, rice may limit the uptake and translocation of OPEs under the influence of the rhizosphere microbiome. Yu studied the transfer and metabolism of TpCP, TmCP, and ToCP in plants and root microorganisms in rice [49]. The results showed that TpCP and TmCP exhibited greater upward transport compared to ToCP, despite sharing identical molecular weights and similar Kow values. The presence of the rhizosphere microbiota promotes rice growth and to some extent inhibits the translocation of TpCP, TmCP, and ToCP into plant tissues. Deng [27] analyzed the concentration and distribution of seven typical OPEs in soils and crops in the urban/suburban city of Chengdu, China. They found that in urban/suburban quarrying soils in Chengdu, the concentration of Σ7OPEs ranged from 91.24 to 544.9 ng/g. Broad bean was found to be easily enriched with TCPP, celery had a relatively high enrichment of TBEP and TEHP, and cowpea was easily enriched with TEHP. Yolanda Picó [28] analyzed wild plants and vegetables from two urban areas in Saudi Arabia for OPE residues, and the concentrations of OPEs in wild plants versus crops on farms were 51.7 ng/g and 13.4 ng/g. Despite the widespread detection of OPEs in environmental media and human samples [50,51,52], their presence in the environment remains unregulated by environmental quality standards (EQS). Meanwhile, it is essential to develop a cost-effective and efficient treatment method to control OPEs, which are stubborn organic pollutants. Advanced oxidation processes (AOP) like electrochemical oxidation, photochemical oxidation, and photocatalysis have shown great potential in eliminating certain persisting organic pollutants [50,51,52]. Exploring ways to degrade OPEs is an effective solution to tackle the pollution problem in the future.

This study focuses on eight common OPEs, namely tris (2-chloroethyl) phosphate (TCEP), tris (1,3-dichloro-2-propyl) phosphate (TDCIPP), triphenyl phosphate (TPHP), 2-ethylhexyl diphenyl phosphate (EHDPP), tris (2-chloropropyl) phosphate (TCPP), tri-o-cresyl phosphate (ToCP), tri-p-cresyl phosphate (TpCP), and tri-m-cresyl phosphate (TmCP). Among them, chlorinated (halogenated) phosphate esters mainly include TCEP, TDCIPP, and TCPP; aryl phosphate esters include TPHP, EHDPP, and TCP. We previously researched agricultural soils and vegetables in the Yangtze River Delta region [53,54]. In this study, we utilized GC-MS/MS to analyze soil and vegetable samples collected from 20 cities along the coast of South China. Simultaneously, we evaluated the potential ecological risks of OPEs and investigated the impact of different physicochemical properties on OPEs. Additionally, we evaluated the health risks associated with OPE ingestion through different routes to provide fundamental data for the study of OPEs in soils and plants in China.

## 2. Materials and Methods

### 2.1. Sample Collection and Preparation

In July 2023, we collected a total of 37 soil and plant samples from agricultural areas in 20 coastal cities in South China. The information regarding the 37 sampling points are presented in Table S1. To ensure that the samples collected are representative, we used the S-shape method to collect the topsoil from a depth of 0 to 10 cm. Additionally, we collected plant samples from various types of leafy vegetables found near the soil samples, including water spinach, pakchoi, sweet potato leaves, and Indian lettuce. By utilizing GPS technology, we were able to accurately record the coordinates of each sampling site. This ensured that our data collection process was precise and reliable. The samples were enclosed in polyethylene kraft paper bags and stored at −40 °C. Subsequently, the samples collected were then freeze-dried using a freeze-dryer (Biocool, Beijing, China) and ground to eliminate any debris. Similarly, plant samples also underwent the process of freeze-drying and grinding. Prior to use, all utensils utilized in this procedure underwent cleaning with deionized water and thorough drying to prevent any potential interference during experimentation.

### 2.2. Chemical Material and Method

Eight OPEs consisting of TCEP, TcPP, TDCIPP, TPHP, EHDPP, ToCP, TmCP, and TpCP were purchased from AccuStandard (New Haven, CT, USA). The dichloromethane, n-hexane, ethyl acetate, and acetone used in the experiment were all HPLC grade; the Silica gel and anhydrous sodium sulfate were activated in advance. The reagents were purchased from Aladdin (Shanghai, China).

### 2.3. Sample Extraction and Purification

The procedures of sample extraction and purification have been described in other references [48] and were used with some modifications. The soil and plant samples were extracted with ultrasonic extraction (Supmile, Jiangsu, China). First, 0.5 g of a plant sample was weighed into a 30 mL glass centrifuge tube and extracted with a mixture of hexane, dichloromethane, and acetone (in a ratio of 2:2:1; *v*/*v*). Then, 20 mL of the extractant was added, and this was shaken well. The samples were then subjected to extraction using an ultrasonic extraction system for 10 min, followed by centrifugation at 4000 rpm for another 10 min. The above procedure was repeated three times. Then, the three supernatants were combined, and we waited for them to purify. Detailed steps for extracting and purifying plant and soil samples and the total organic carbon (TOC) and pH assays are provided in Text S1.

### 2.4. Instrumental Analysis

The quantification of OPEs was conducted using a GC-MS/MS (7890A/7000C, Agilent Technologies, Santa Clara, CA, USA) with an HP-5MS quartz capillary column (30 m × 0.25 mm inner diameter × 0.25 μm film thickness). The mass spectrometer used electron bombardment (EI) for ionization, allowing quantitative analysis through ion monitoring (SIM) mode. Detailed information on the ions used for quantification is available in Table S2. The procedure starts by setting the initial temperature to 50 °C for 2 min, then gradually increasing it to 300 °C at a rate of 25 °C per minute and maintaining it at that temperature for 5 min. The transfer line, ion source, and quadrupole temperatures were set at 290, 280, and 150 °C, respectively.

### 2.5. Ecological Risk and Human Health Risk Assessment

OPEs are typical endocrine disruptors that are widely present in the ecological environment with the continuous advancement of industrialization and urbanization. Soil is a crucial natural resource that serves as the foundation for agriculture and a reservoir for pollutants. The lipophilicity, hydrophobicity, and chemical stability of OPEs make them easily adsorbed in soil [55]. Furthermore, this may pose a threat to the ecological environment and human health through the food chain or other means. Therefore, it is important to conduct timely ecological and human health risk assessments to evaluate their impacts on the ecosystem. The ecological risk assessment in this study followed the methodology outlined by Yadav [16]. In accordance with a model recommended by the US EPA (2011) [56], we conducted an assessment of the human health risks associated with OPE exposure to elucidate the impacts of OPE on human health.

#### 2.5.1. Ecological Risk Assessment

The risk quotient (RQ) method was employed to assess whether the identified levels of OPEs in soil samples would present a risk to environmental targets. The calculation formula is as follows:

$$RQ=\frac{MEC}{{PNEC}_{soil}}$$

The RQ is the ratio of the measured environmental concentration (MEC) to the predicted ineffective concentration (PNEC). The PNEC value for OPEs in soil is derived from data presented in previous literature and reports, as depicted in Table S3. The risk assessment was only performed on chemicals for which the EC50 values were available. The maximum potential ecological risk was evaluated based on common standards: an RQ < 0.1 indicates a lower risk of potential adverse reactions; an 0.1 ≤ RQ < 1 indicates a moderate risk of potential adverse reactions; and an RQ ≥ 1 indicates a higher risk of potential adverse reactions.

#### 2.5.2. Human Health Risk Assessment

Soil pollution risks are categorized into non-carcinogenic and carcinogenic risks, quantified by the hazard quotient (HQ) and carcinogenic risk (CR). The HQ quantifies non-carcinogenic risks, while the CR measures carcinogenic risks. Pollutants in surface soil can pose health risks to humans through ingestion, skin contact, and inhalation pathways. To determine safe exposure levels, we calculated chronic daily intake (CDI) via these pathways using the following formula:

$${CDI}_{ingest}=\frac{C*IR*CF*ED*EF}{BW*AT}$$

$${CDI}_{dermal}=\frac{C*CF*SA*AF*ABS*ED*EF}{BW*AT}$$

$${CDI}_{inhale}=\frac{C*HR*ED*EF}{PEF*BW*AT}$$

To calculate the hazard quotient (HQ), divide the chronic daily intake (CDI) by the reference dose (RfD). When evidence supporting the interaction of OPEs risk is lacking, the total hazard quotient (THQ) can be computed by summing the HQ values of all OPEs:

$$HQ=\frac{{CDI}_{ingest}}{RfD}+\frac{{CDI}_{dermal}}{RfD*GIABS}+\frac{{CDI}_{inhale}}{RfC}$$

$$THQ=\sum {HQ}_{x}$$

To compute cancer risk (CR), multiply the chronic daily intake (CDI) by the respective cancer slope factor (SFO) for each exposure pathway. Then, sum up the CR values of all OPEs to obtain the total cancer risk (TCR):

$$CR={CDI}_{ingest}*SFO+{CDI}_{dermal}*\frac{SFO}{GIABS}+{CDI}_{inhale}*IUR$$

$$TCR=\sum {CR}_{x}$$

$$EDI=\frac{\sum_{i=1}^{n}Ci\times DCi}{BW}$$

Prior research failed to assess the inhalation risks associated with OPE exposure due to a scarcity of Reference Concentration (RfC) and Inhalation Unit Risk (IUR) data. Therefore, in this study, the health risk assessment was not conducted on exposure to OPEs through inhalation routes. The parameters for calculating ecological risk, non-carcinogenic risk, and carcinogenic risk are listed in Tables S3 and S4.

For plants, we referred to the risk assessment method adopted by Zhang [57] to assess the risk of OPE exposure to crops in the region. We estimated the daily intake of OPEs through consuming crops using the above formula. Food consumption rates (DC) were established using data from various nationwide surveys conducted in China. [58,59]. BW refers to body weight, and the specific parameters can be found in Table S5, as per the data from USEPA2011 [56].

### 2.6. Quality Assurance and Quality Control

The limits of quantification (LOQs) were estimated on the basis of a signal-to-noise ratio of 10. The LOQs for eight OPEs in soils were 0.003–0.006 ng/g, and in plants were 0.08–0.13 ng/g.

### 2.7. Data Processing and Analysis

Statistical analyses were conducted using various software, including Microsoft Excel 2016, Origin 10.6 (OriginLab Inc., Northampton, MA, USA), and SPSS 22.0 (Armonk, IBM, New York, NY, USA). The spatial distribution maps of 8 types of OPEs in soil and plants were mapped using ArcGIS 10.5. Pearson’s correlation analysis was utilized to assess the relationship among OPE concentrations, TOC, and the pH value of the soil.

## 3. Results and Discussion

### 3.1. General Characteristics of OPE Concentrations in Soil

Table 1 presents descriptive statistics for OPEs in 37 soil samples collected from coastal areas of South China. In this study, OPEs were found in all soil samples, indicating their widespread presence in the natural environment. The concentration range of the eight OPEs in the soil was 74.7–410 ng/g, and the mean value was 255 ng/g. Among the eight OPEs, the detection rate of TCEP, TcPP, and TDCIPP was 100%, and the detection rates of TPHP, EHDPP, ToCP, TpCP, and TmCP were 97.3%, 78.3%, 83.8%, 73%, and 86.5%, respectively. The extent of the contribution of each OPE in the soil is shown in Figures S1 and S2. Out of the 37 soil samples, TDCIPP had the highest concentration, contributing 12.30–77.65% with a mean value of 51.84%, followed by TcPP, contributing 3.20–59.80% with a mean value of 24.37%, and TCEP, TPHP, EHDPP, ToCP, TpCP, and TmCP contributed 1.73–42.65%, 0–6.85%, 0–24.28%, 0–15.12%, 0–15.23%, and 0–12.28%, with mean values of 10.20%, 3.44%, 2.42%, 2.48%, 2.35%, and 2.90%, respectively. Among the 20 coastal cities investigated in this study, 16 of them had total OPE concentrations higher than the mean value of 225 ng/g in this study, indicating that a certain degree of pollution by OPEs was present in all cities. The average concentrations of the eight OPEs were TDCIPP > TCPP > TCEP > TPHP > ToCP > TmCP > EHDPP > TpCP, with chlorinated OPEs having higher concentrations than arylated OPEs. The presence of OPEs in soil from various regions and countries has been researched and compiled. Nepal is facing significant environmental pollution issue due to the lack of advanced pollution control technologies. The accumulation of significant amounts of consumer goods and construction materials in urban areas leads to high concentrations of OPEs, as proper disposal options are lacking. Σ8OPE concentrations in soil from four cities in Nepal [42] ranged from 24.9 to 2.79 × 10^4^ ng/g, with Kathmandu exhibiting the highest contamination level at a median concentration of up to 662 ng/g. The maximum concentration of OPEs in the urban soils of Bursa, Turkey, reached 468 ng/g [60]. This is mainly due to the high level of industrialization in the region. In China, the soil concentration of OPEs in Dalian was 1.07–288 ng/g [61], with a mean value of 4.23 ng/g. The concentration of OPEs in the soil in Ningbo [62] ranged from 162.7 to 986.0 ng/g, with an average value of 469.3 ng/g. In Guangzhou [63], the concentration of OPEs in the soil ranged from 41 to 1370 ng/g, with an average value of 250 ng/g. The higher contamination of OPEs in this area was primarily attributed to the presence of production enterprises and electronic waste treatment plant aggregation related to OPEs [64]. Elevated concentrations of soil OPEs in urban areas as a result of extensive industrial activities combined with frequent agricultural activities can also aggregate OPEs in agricultural fields. Concentrations of OPEs detected in agricultural soils in the Three Gorges Reservoir area [65] in China were in the range of 52.1–680 ng/g, with a mean value of 266 ng/g. In agricultural soils in Zhejiang [66], these concentrations were in the range of 9.15–132 ng/g, with a mean value of 24.9 ng/g, and in agricultural soils in Jinan [67], the mean value of this parameter, 7.89 ng/g, falls within the range of 2.55 to 17.6 ng/g. Detailed figures are shown in Figure S3. The concentration distribution of OPEs in farmland soils varies across regions. In this study, the concentrations of OPEs ranged from 74.7 to 410 ng/g, averaging 255 ng/g, which is comparable to those found in farmland soils in the Three Gorges Reservoir area of China but higher than those in farmland soils in Zhejiang and Jinan.

By analyzing the sampling sites, it was found that the soil sampling points with higher OPE concentrations were located near automobile repair factories, electrical appliance companies, and furniture companies. OPEs in agricultural soils mainly originate from remote atmospheric transport, atmospheric deposition, air–soil exchange, sewage irrigation, and mulch film application. OPEs in cities enter the soil environment through remote atmospheric transport, atmospheric deposition, and air–soil exchange, and are then adsorbed to the soil [68,69,70,71,72]. Irrigation of sewage containing OPEs is prone to adversely affect agricultural soils [73,74,75], and at the same time, mulch film [76,77] is also oxidized into OPEs in the soil environment, which is then adsorbed by the soil and causes contamination of agricultural soils. The above routes all have an impact on the concentration of OPEs in farmland soil to some extent. In this study, it is speculated that the main sources of OPEs in farmland soil are the diffusion and transportation of urban air and dust, rainfall deposition, irrigation water, and plastic film application.

Of the individual OPEs, TDCIPP, TcPP, and TCEP are the primary residues of the eight OPEs in the studied agricultural soils. The concentration of TDCIPP varied from 16.2 to 245 ng/g, averaging 139 ng/g. The concentration of TcPP varied from 6.17 to 106 ng/g, averaging 55.5 ng/g, and the concentration of TCEP ranged from 3.99 to 154 ng/g, with a mean value of 28.3 ng/g. Some studies have investigated the presence of OPEs in soil: Zhang [78] conducted a study on OPEs in farmland soils of Heilongjiang, Henan, Hubei, and Guangxi. The study found that the average concentration of TCEP was 17.9 ng/g, TcPP was 3.59 ng/g, and TDCIPP was 2.68 ng/g. Wang [79] found that TCEP concentrations in soils across China were relatively higher than in other OPEs, with a minimum value of 0.014 ng/g and a maximum value of 182 ng/ng. Li [66] revealed that TCEP concentrations ranged from 0.321 to 4.96 ng/g in agricultural soils in Zhejiang. Sang [80] discovered concentrations of TCEP, TcPP, and TDCIPP in Beijing’s greenhouses, specifically 0.11 ng/g for TCEP, 6.03 ng/g for TcPP, and 1.17 ng/g for TDCIPP. In this study, the concentrations of TCEP, TcPP, and TDCIPP were significantly higher than those mentioned above. The variations in electronic factory distributions, economic levels, temperatures, and agricultural crop planting and cultivation habits contribute to these differences. TDCIPP, TCEP, and TcPP are the most commonly utilized chlorinated OPEs in flexible and rigid polyurethane foam (PUF) materials [18] as well as in building materials and furniture products. Exogenous contaminating chemicals are usually eliminated in environmental media mainly by two pathways: physicochemical transformation (photodegradation) and biotransformation (biodegradation). For OPEs in environmental media, water-based studies have shown that light degrades alkyl organophosphates (TBOEP, TiBP, and TnBP), but chlorine-containing TCIPP, TCEP, and TDCIPP cannot be eliminated by photodegradation [81,82]. In addition, Kawagoshi [83] showed that OPEs are difficult to biodegrade via biodegradation in both aerobic and anaerobic environments, and thus TCEP and TDCIPP are more likely to accumulate in high concentrations in soil. As additive flame retardants, OPEs can be easily adsorbed into different environmental media and then enter the soil in different ways, and automobile repair shops, electrical appliance companies, and furniture companies in the vicinity of sampling sites may provide a sufficient source of OPEs.

In this study, eight OPEs were mainly categorized into chlorinated OPEs as well as aryl OPEs, with concentrations of chlorinated OPEs ranging from 55.4 to 383 ng/g, with a mean value of 223 ng/g, and concentrations of aryl OPEs ranging from 6.80 to 99.7 ng/g, with a mean value of 32.7 ng/g. Chlorinated OPEs, such as TCPP and TCEP, are commonly found in sewage treatment plant wastewater, stormwater, roadway runoff, surface water, and indoor/outdoor dust as the major OPE species [84,85,86,87]. In addition, the concentration of TCEP is typically the third highest in soil, as a result of its high concentration and persistence in media such as stormwater and dust [16,72]. Alkylated or chlorinated OPEs have been reported to dominate soils in Bursa, Turkey [60]. These reasons may explain why chlorinated OPEs had a higher abundance in our study.

### 3.2. Regional Differences in OPE Contamination

**Figure 1:**
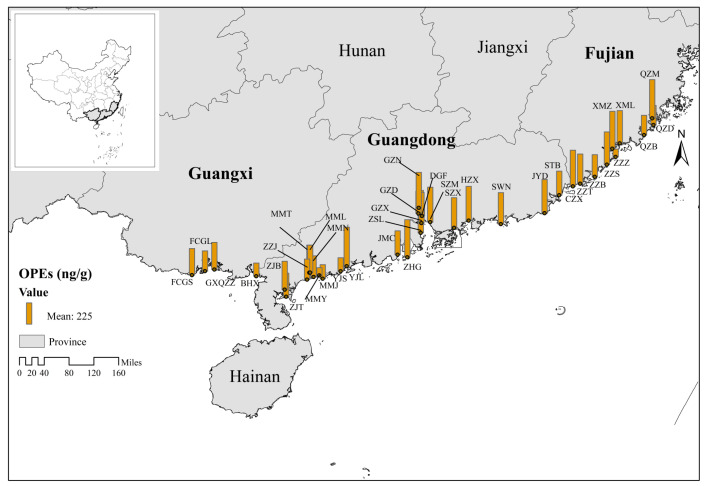
Spatial distribution of OPEs in soils from 20 coastal cities in South China.

**Figure 2:**
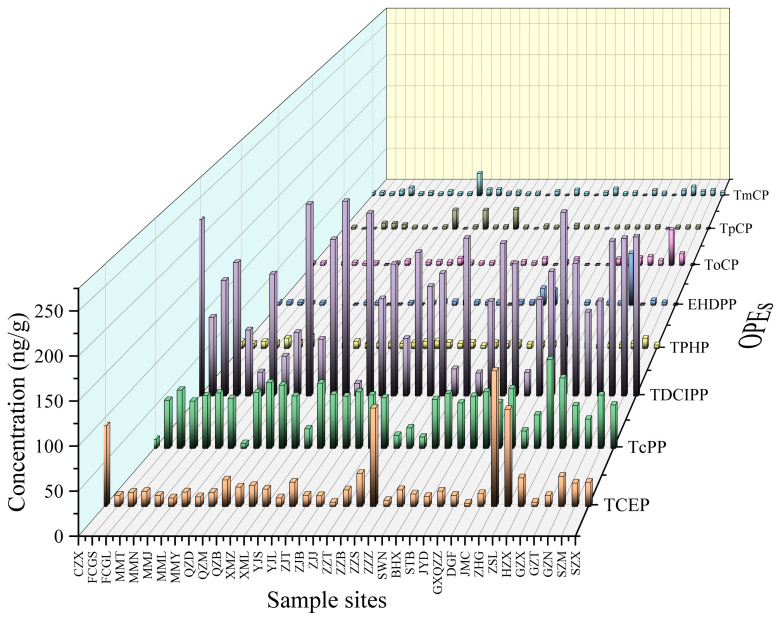
Concentration of eight OPEs in soils from 20 coastal cities in South China.

There exist certain regional differences in the pollution attributes of OPEs due to the relationship between urban activities and agricultural cropping patterns as OPE sources. Figure 1 and Figure 2 display the spatial distribution of ∑8OPE concentrations in order to visualize the concentration distribution. On average, the highest concentration of ∑8OPEs in agricultural soil was in Guangdong Province at 262 ng/g, followed by Fujian Province with 258 ng/g, and the lowest level was in the Guangxi Zhuang Autonomous Region at 211 ng/g. As shown in Figure 1 and Figure 2, a sampling site in Zhongshan City, Guangdong Province, had the highest concentration of ∑8OPEs, which was mainly attributed to the fact that the site was surrounded by companies related to electrical appliances, furniture, and textiles, and all of them were near farmlands with large planting areas, extensive coverage, and frequent agricultural activities. TOC and pH are important properties of solid substrates in the environment, which have a significant effect on the adsorption, transport, and transformation of OPEs [88,89,90]. We determined the soil TOC content and pH of 37 sampling sites. The results revealed a range of TOC content, spanning from a minimum of 0.41 g/kg to a maximum of 36.0 g/kg, with an average of 10.5 g/kg. Similarly, the pH values ranged from a minimum of 5.13 to a maximum of 8.85, with an average of 6.59. The correlations between the eight OPEs and TOC and pH were analyzed using Pearson’s correlation coefficient. The results revealed weak positive correlations between the eight OPEs and total organic carbon as well as pH; however, these correlations did not reach statistical significance (*p* > 0.05). This suggests that OPEs are not correlated with the organic matter content of soil. This could be attributed to their limited binding capacity with organic matter in soil, which depends on their physical properties and direct interactions.

OPEs are the main contaminants detected in furniture, textiles, and electronics. As additive flame retardants, OPEs are easily volatilized and leached out during production and transportation and then transported through the atmosphere or deposited into agricultural soils. Wastewater from furniture factories and textile factories is discharged without proper treatment and enters agricultural soil through irrigation water [73,74,75]. Meanwhile, the application of mulch film is also a significant source of OPEs in agricultural soils. Mulch film, primarily composed of plastic, contains organophosphate antioxidants that can oxidize into OPEs in soil environments [3,76,77], exacerbating OPE contamination. Furthermore, the concentration of OPEs is notably high in e-waste recycling areas such as Shenyang [91], as well as in urban areas across China. Conversely, the presence of OPEs in agricultural soils is relatively low. The primary reason for the high pollution levels may stem from the rapid expansion of local electronics and furniture industries, leading to significant emissions of OPEs from factories in the area. The relatively high concentration of OPEs in the coastal region of South China when compared to other regions can be attributed to regional variations in the siting of factories, development, and economic and agricultural activities, as well as regional differences in soil types at specific geographic locations. It is crucial that we pay attention to these factors, even if a comprehensive study of OPEs in agricultural soils in the Pearl River Delta has not yet been carried out. Being mindful of these aspects can help us make informed decisions and promote sustainable agriculture in the region. Through a correlation analysis of the major individual OPEs in soil, it was found that the concentrations of TcPP, TCEP, and TDCIPP were positively associated, but not significantly (*p* > 0.01).

### 3.3. Overall Characteristics of OPE Contamination in Plants

Table 2 presents the descriptive statistics for the individual and total concentrations of OPEs in plants (vegetables) collected from 37 sampling sites in the coastal areas of South China. The total concentrations of the eight OPEs varied from 202 to 751 ng/g, with an average concentration of 381 ng/g. OPEs were detected in all plant samples, among which TCEP, TcPP, and TPHP were detected at a rate of 100%, followed by TDCIPP at a rate of 97.3%, and the detection rates of the other OPEs were below 90%. The degree of contribution of each OPE in plants is shown in Figure S1. Among the 37 plant samples, in agreement with the soil samples, the TDCIPP concentration was the highest among the eight OPFRs, with values in the range of 0–57.42% and a mean value of 35.31%, followed by TcPP with 5.49–55.46% and a mean value of 29.03%; ToCP, TCEP, TPHP, EHDPP, TmCP, and TpCP contributed 0–51.75%, 1.83–37.34%, 0.77–22.59%, 0–30.79%, 0–47.04%, and 0–1.78%, with mean values of 11.06%, 8.22%, 2.93%, 6.57%, 6.01%, and 0.67, respectively, and TDCIPP, TcPP, and ToCP accounted for 75.4% of the eight OPEs, suggesting that they are the main OPEs in coastal plants in southern China. The statistical analysis indicated no significant correlation between soil OPEs and plant OPEs. Furthermore, it was found that, in contrast to agricultural soils, the accumulation of OPEs in plants was noticeably higher.

Some studies on OPEs in plants have been conducted in China as well as in other countries. Vegetables from China [57], generally contained high levels of OPEs, with an average mean total OPE concentration of 19.35 ng/g, of which TEHP was the highest, with an average of 12.7 ng/g, followed by TCEP, with an average of 4.16 ng/g, which was 1–2 times higher than that from vegetables in Sweden, Belgium, and Australia. This is because the levels of OPEs in water and soil in China are generally higher than those in the aforementioned three countries. Moreover, most OPEs have high water solubility, resulting in increasing accumulation in vegetables from water and soil. TPHP (with an average value of 2.01 ng/g ww) and TCIPP (with an average value of 1.91 ng/g ww) were identified as the predominant OPEs in Belgian and Australian vegetables, respectively. Their concentrations were discovered to exceed those of other OPEs by one to two orders of magnitude [92,93]. In a separate study conducted by Poma in Uppsala, Sweden, it was found that the average concentration of TPHP (0.07 ng/g ww) was significantly lower than the average concentrations of TCEP (0.41 ng/g ww), TDCIPP (0.37 ng/g ww), EHDPP (0.28 ng/g ww), and TCIPP (0.18 ng/g ww) [94]. In addition, green leafy vegetables such as Chrysanthemum coronarium, cauliflower heart, and leeks contain higher levels of OPEs than stem or root vegetables, which may be because these vegetables have a larger area of wax and cuticle and are therefore more likely to be enriched with OPEs [57]. In comparison to results from other research areas, the levels of Σ8OPEs in plants from the coastal region of southern China were typically significantly higher. Differences in the ability of various plant species to accumulate OPEs, as well as variances in time periods and geographic locations, could be the cause of the dissimilarity.

The monomers of OPEs in vegetables were largely consistent with those of OPEs in soil, as shown in Figure S4. TDCIPP, TcPP, and ToCP were the main OPEs in the plants, with an average content of 129 ng/g, 102 ng/g, and 49.5 ng/g, respectively. TDCIPP, TcPP, and TCEP are frequently employed as chlorinated organophosphate esters (OPEs) in the manufacturing of consumer goods, including plastics, electronics, textiles, furniture foams, construction materials, and automotive interiors. The other OPEs in this study are non-halogenated OPEs, which are mainly used as plasticizers, lubricants, defoamers, and other additives, except some are used as flame retardants. Chlorinated OPE levels may be higher due to the presence of electronic, textile, and furniture companies operating near the sampling site for an extended period. Conversely, the limited uptake of non-chlorinated OPEs by plant roots and their low accumulation in soil can be attributed to variations in physical properties, such as hydrophilicity.

**Figure 3:**
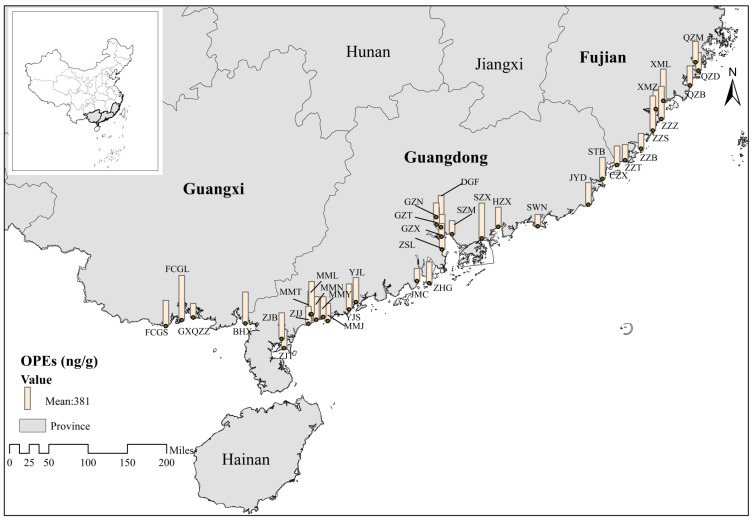
Spatial distribution of OPEs in plants collected in 20 cities along the coast of South China.

**Figure 4:**
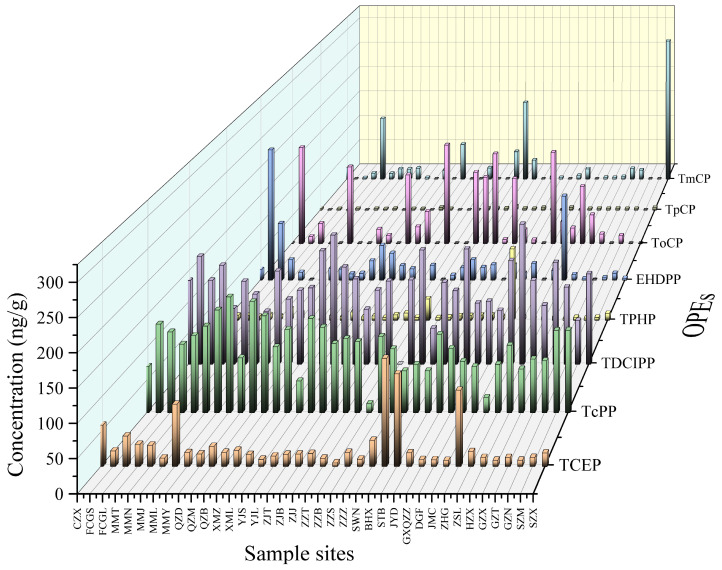
Concentration of eight OPEs in plants collected in 20 cities along the coast of South China.

OPEs in plants are mostly caused by soil and air deposition and irrigation with sewage, and they are intimately associated with both industrial and agricultural processes. Also demonstrating geographical specificity are the pollution features of OPEs in plants. Figure 3 and Figure 4 depict the regional distribution of OPEs in plants in South China’s coastline region. The results were different from those of the spatial distribution of soil, with the highest average concentration of OPEs in plants in the Guangxi Zhuang Autonomous Region, 486 ng/g, followed by Fujian Province, 386 ng/g, and lastly Guangdong Province, 363 ng/g. The regional differences between plants and soils may be due to the differences in the crop types and planting environments, but there are few studies on the relationship between OPEs in plant–soil systems in different regions. Therefore, it is difficult to compare our results with other studies, and further research on the mechanism is needed in the future.

A strong association (*p* < 0.05) was found between TDCIPP and ToCP in the main OPEs (TDCIPP, TcPP, ToCP, and TCEP) in coastal agricultural plants in South China. This implies that some OPEs might have comparable input paths and sources. The overall content of OPEs in South China’s coastal soils and plants did not differ significantly (*p* > 0.05). This may be attributed to the complexity of the origins of OPEs in plants, as they can potentially absorb these compounds from sources other than soil.

### 3.4. Ecological Risk and Human Risk Assessments of OPEs

Since there is no international environmental health standard for OPEs, there are few reference data available, and in this study, only some relevant data are available for TCEP, TDCIPP, TPHP, and EHDPP, so the following ecological risk assessment is performed only for the four OPEs mentioned above, and the non-carcinogenic risk assessment is performed only for TPHP, TCEP, and TDCIPP in soil, the carcinogenic risk assessment is performed only for TCEP and TDCIPP, and the EDI calculations are performed only for TCEP, TDCIPP, and TPHP in plants.

**Figure 5:**
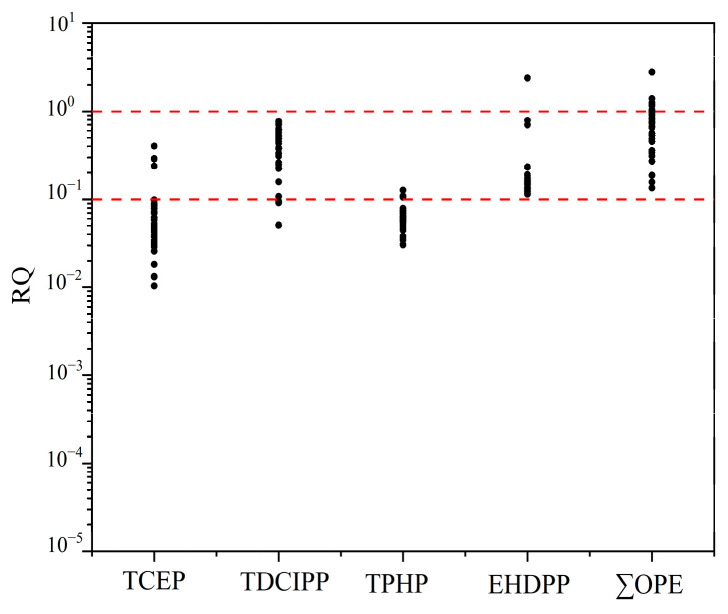
Ecological risks of each monomer of OPEs and total OPEs in the coastal areas of South China.

The ecological risk assessment of OPEs in the soil of the coastal areas of South China was calculated using a risk quotient (RQ), and the detailed results are shown in Table S6. Based on the obtained RQ value, the combined toxicity of OPEs in the soil was evaluated by adding up each substance at each sampling point. The findings indicated that the aggregate RQ values for the four OPEs fell within the range of 0.13 to 2.77. Among them, 21.6% of the soil had a total RQ value greater than 1, indicating high risk, while the rest was within the range of 0.1 to 1, indicating moderate risk. The 37 sampling points are all located in high-risk or medium-risk areas, all of which are economically developed or concentrated areas with frequent human activities, indicating that frequent human activities and rapid economic development pose potential threats to the ecosystem. Figure 5 shows that only one sampling point for EHDPP had an RQ exceeding 1, indicating high ecological risk, while for TCEP, TDCIPP, and TPHP, 10.8%, 89.2%, and 78.4% of the points, respectively, had RQs ranging from 0.1 to 1, indicating moderate risk.

Due to the lack of reliable toxicity data, health risks were evaluated for only some of the OPEs. Moreover, since we only have parameters related to the ingestion and dermal contact of OPEs, we did not conduct carcinogenic and non-carcinogenic risk assessments for other pathways. The non-carcinogenic risk of soil was calculated for a total of three OPEs based on the available RfD values, as shown in Table S7A, and the total HQs for the three compounds ranged from 7.55 × 10^−3^ to 0.15 and 1.96 × 10^−3^ to 2.74 × 10^−2^ for children and adults, with mean values of 3.90 × 10^−2^ and 1.01 × 10^−2^, respectively. The HQ values for children were found to be higher than those for adults, indicating that children may be more vulnerable to non-carcinogenic risk threats compared to adults. However, both HQ values fell below 1, indicating a low non-carcinogenic risk posed by OPEs in the soil of the coastal region of South China.

**Figure 6:**
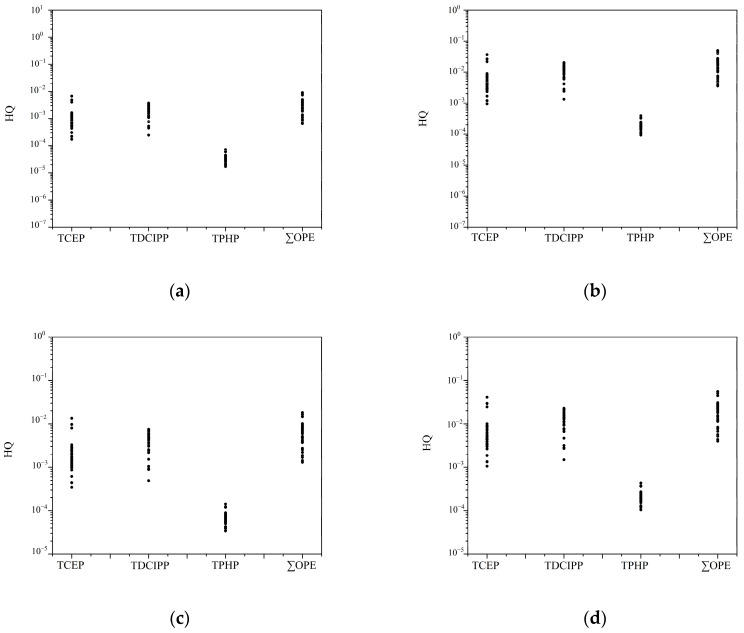
Hazard quotient (HQ) was employed to assess non-carcinogenic risk posed by OPEs in soils. Data points on graph represent the HQ values at each sampling site. Object: adult; pathway: ingestion (**a**). Object: children; pathway: ingestion (**b**). Object: adult; pathway: dermal contact (**c**). Object: children; pathway: dermal contact (**d**).

**Figure 7:**
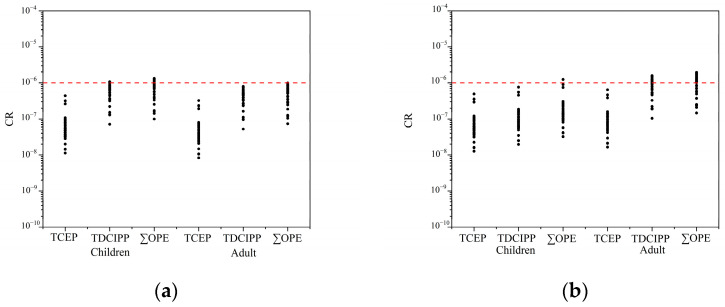
Carcinogenic risk values (CR) were employed to assess potential carcinogenic risk due to TCEP and TDCIPP in soils. Data points on graph represent CR values at each sampling site. Object: children and adults; pathway: ingestion (**a**). Object: children and adults; pathway: dermal contact (**b**). The auxiliary lines with CR values of 10^−6^.

In terms of the individual compounds, as depicted in Figure 6 and Table S7B–D, TCEP (children 1.42 × 10^−2^, adults 3.69 × 10^−3^), TDCIPP (mean value: children 2.44 × 10^−2^, adults 6.35 × 10^−3^), and TPHP (mean value: children 3.98 × 10^−4^, adults 1.04 × 10^−4^), with the highest HQ for TDCIPP, were found, followed by TCEP and TPHP. Among the different modes of exposure, for TCEP (ingestion: 6.70 × 10^−3^ for children and 1.23 × 10^−3^ for adults; dermal contact: 7.50 × 10^−3^ for children and 2.46 × 10^−3^ for adults), TDCIPP (ingestion: 1.15 × 10^−2^ for children and 2.12 × 10^−3^ for adults; dermal contact: 1.29 × 10^−2^ for children and 4.23 × 10^−3^ for adults), and TPHP (ingestion: 1.88 × 10^−4^ for children and 3.46 × 10^−5^ for adults; dermal contact: 2.10 × 10^−4^ for children and 6.90 × 10^−5^ for adults), the risk of exposure to OPEs through dermal contact was higher than that of soil ingestion. This suggests that dermal contact serves as the primary exposure route. This result is consistent with previous risk assessments of soil [95,96]. The carcinogenic risk assessment of TCEP and TDCIPP in soil is shown in Figure 7 and Table S8. The total CR values for children and adults ranged from 1.74 × 10^−7^ to 2.38 × 10^−6^ and 2.19 × 10^−7^ to 2.94 × 10^−6^, with mean values of 9.22 × 10^−7^ and 1.53 × 10^−6^, respectively. CR values falling below 10^−6^ are classified as very low carcinogenic risk, while those ranging between 10^−6^ and 10^−4^ are considered low, between 10^−4^ and 10^−3^ are classified as medium, between 10^−3^ and 10^−1^ as high, and those exceeding 10^−1^ as very high carcinogenic risk. The adult group has more carcinogenic risk than children, which may be since adults have been ingesting pollutants for a longer period than children. Overall, the CR values for all samples range between CR < 10^−6^ or 10^−6^ < CR < 10^−4^, indicating a low carcinogenic risk for both children and adults.

Due to limited studies on the health risks of OPEs in plants, we assessed their potential threat to humans by calculating the dietary intake of OPEs through food consumption. This intake was then compared to the reference dose (RfD) (ng/kg bw/day). Table 3 shows the dietary intake data of OPEs in children and adults.

The estimated daily intake (EDI) range for total children OPEs varied from 239.46 to 1708.1 ng/kg bw/day, with an average of 984.03 ng/kg bw/day. For total adult OPEs, the EDI range was between 244.80 and 1746.1 ng/kg bw/day, averaging 1005.9 ng/kg bw/day. Among individual OPEs, TDCIPP and TCEP exhibited the highest exposure doses. Comparing the EDI values of OPEs with their reference doses per day (RfDs), RfDs were calculated by dividing the chronic NOAEL (no observed adverse effect level) by 1000 (safety factor). In this study, three compounds were exposed at levels lower than their RfD values. Although the exposure levels of several compounds mentioned above were lower than their RfD values, the mean EDI value for adults in the study of Zhang [57] was 539 ng/kg bw/day, which was double that in the present study and deserves serious attention.

To our knowledge, there is limited literature on OPE concentrations in foods. As a result, this study may underestimate the risk assessment of the selected OPEs due to the small number analyzed, with other potential OPEs in foods not considered. Therefore, we cannot disregard the potential threat of exposure to mixed OPEs, despite the observed levels being considerably lower than their respective RfD values.

## 4. Conclusions

This study presents an assessment of OPE contamination in crops and agricultural soils in the South China coastal region. OPEs were detected at 37 sampling sites in both farmland soils and crops, with concentrations exceeding those in other regions. This suggests that rapid urbanization and frequent farming in coastal South China might have led to more OPEs building up in the environment. It is worth noting that the concentrations of OPEs in plants were higher than in soil, indicating a potential trend of OPE accumulation in plants over time, likely attributed to bioaccumulation. The ecological risk assessment of 37 sampling sites predominantly indicated high or medium risks. However, both non-carcinogenic and carcinogenic assessments indicated low risks associated with organophosphate esters (OPEs) in the soil along South China’s coastal region. Notably, dermal contact emerged as the primary exposure route. EDI calculations showed that, although the exposure level of OPEs was relatively low in comparison with the RfD, it was twice as high in the present study compared with the other studies. Excessively high concentrations of OPEs require a comprehensive understanding of their environmental impacts and potential threats to human health. Future research should prioritize reducing OPE exposure in plants consumed in diets to ensure agricultural safety and ecosystem health. We recommend planting crop varieties with low OPE accumulation to minimize the bioaccumulation of OPEs in crops. Meanwhile, in recent years, microplastics (MPs) have received widespread attention as an increasingly serious environmental problem [97], and OPEs, as flame retardants and plasticizers, are closely related to the existence of microplastics. Xing [98] researched how different sizes and amounts of polystyrene MNPs affect the absorption of eight types of OPEs in rice seedlings through hydroponic experiments. The findings revealed that MNPs can change how OPEs are absorbed, transported, and stored in rice seedlings. However, there are not yet many studies about how MNPs take in and store OPEs in soil and plants. We will focus on this in the future to continue our research.

## Figures and Tables

**Table 1:** Concentrations of Σ8OPEs (ng/g, dw) were measured in 37 agricultural soil samples located in South China’s coastal region.

Compound	Detection Rate (%)	Mean	Median	Min	Max
TCEP	100	28.3	16.7	3.99	154
TcPP	100	55.5	62.9	6.17	106
TDCIPP	100	139	154	16.2	245
TPHP	97.3	7.96	7.57	-	16.5
EHDPP	78.3	6.30	4.04	-	71.9
ToCP	83.8	6.76	4.85	-	51.8
TpCP	73.0	5.23	3.44	-	30.3
TmCP	86.5	6.43	5.26	-	35.2
Σ8OPEs	100	255	264	74.7	410

Note: “-”, below the limit of quantification.

**Table 2:** Concentrations of Σ8OPEs (ng/g, dw) in 37 vegetable samples located in South China’s coastal area.

Compound	Detection Rate (%)	Mean	Median	Min	Max
TCEP	100	30.9	19.1	5.47	157
TcPP	100	102	105	14.1	177
TDCIPP	97.3	129	134	-	226
TPHP	100	11.3	7.05	2.94	121
EHDPP	83.8	29.3	19.3	-	231
ToCP	64.9	49.5	18.0	-	182
TpCP	72.2	2.44	2.70	-	7.44
TmCP	64.9	26.1	5.16	-	278
Σ8OPEs	100	381	350	202	751

Note: “-”, below the limit of quantification.

**Table 3:** Estimated daily dietary intakes (EDI, ng/kg bw/day) of OPEs and corresponding RfD values.

Compound	Children			Adult			RfD ng/kg bw Day
	Max	Min	Mean	Max	Min	Mean	
TCEP	904.12	31.360	177.53	924.27	32.060	181.49	22,000
TDCIPP	1296.2	0.0000	741.75	1325.1	0.0000	758.28	15,000
TPHP	694.93	16.850	64.740	710.41	17.230	66.180	70,000
Σ3OPEs	1708.1	239.46	984.03	1746.1	244.80	1005.9	na ^a^

^a^ RfD value is not available.

## Data Availability

Data are contained within the article.

## Supplementary Materials

The following supporting information can be downloaded at: https://www.mdpi.com/article/10.3390/toxics12040286/s1, Text S1: The method of soil sample extraction and detection of TOC and pH in soil; Table S1: Details of the 37 sampling sites; Table S2: GS-MS/MS parameter of OPEs. Table S3: Parameters for calculating PNECsoil, RfD, and SFO of OPEs in soils; Table S4: Calculation parameters and values used in the health risk assessment model to evaluate exposure risks of OPEs in soils; Table S5: Daily consumption (g/day) and body weight (kg) for general Chinese children and adults; Table S6: Estimated risk quotient (RQ) of different OPEs for soil and total RQ in the coastal areas of South China; Table S7: The hazard quotient (HQ) for different OPEs with two pathways in soils; Table S8: The carcinogenic risk values (CRs) for different OPEs with two pathways in soils. Figure S1. Contribution degree of each OPE monomer in soil and plants; Figure S2. Concentrations of each OPE in the soil; Figure S3. The concentration and geographical distribution of OPEs in soils in coastal areas of South China and other studied regions; Figure S4. Concentrations of each OPE in plants [99,100,101,102,103,104,105].

## References

[1] Wei G.L., Li D.Q., Zhuo M.N., Liao Y.S., Xie Z.Y., Guo T.L., Li J.J., Zhang S.Y., Liang Z.Q. (2015). Organophosphorus flame retardants and plasticizers: Sources, occurrence, toxicity and human exposure. Environ. Pollut..

[2] Hou M.M., Shi Y.L., Na G.S., Cai Y.Q. (2021). A review of organophosphate esters in indoor dust, air, hand wipes and silicone wristbands: Implications for human exposure. Environ. Int..

[3] Blum A., Behl M., Birnbaum L.S., Diamond M.L., Phillips A., Singla V., Sipes N.S., Stapleton H.M., Venier M. (2019). Organophosphate ester flame retardants: Are they a regrettable substitution for polybrominated diphenyl ethers?. Environ. Sci. Technol. Lett..

[4] Li T.Y., Bao L., Wu C.C., Liu L.Y., Wong C.S., Zeng E.Y. (2019). Organophosphate flame retardants emitted from thermal treatment and open burning of e-waste. J. Hazard. Mater..

[5] Zhang R.J., Yu K.F., Li A., Zeng W.B., Lin T., Wang Y.H. (2020). Occurrence, phase distribution, and bioaccumulation of organophosphate esters (OPEs) in mariculture farms of the Beibu Gulf, China: A health risk assessment through seafood consumption. Environ. Pollut..

[6] Yadav I.C., Devi N.L. (2020). Data on fate and distribution of organophosphate esters in the soil-sediments from Kathmandu Valley, Nepal. Data Brief.

[7] Luo Q., Gu L.Y., Wu Z.P., Shan Y., Wang H., Sun L.N. (2020). Distribution, source apportionment and ecological risks of organophosphate esters in surface sediments from the Liao River, Northeast China. Chemosphere.

[8] Guo J., Li Z., Ranasinghe P., Rockne K.J., Sturchio N.C., Giesy J.P., Li A. (2020). Halogenated flame retardants in sediments from the Upper Laurentian Great Lakes: Implications to long-range transport and evidence of long-term transformation. J. Hazard. Mater..

[9] Zhao L.M., Zhang Y.Y., Deng Y.R., Jian K., Li J.H., Ya M.L., Su G.Y. (2020). Traditional and emerging organophosphate esters (OPEs) in indoor dust of Nanjing, eastern China: Occurrence, human exposure, and risk assessment. Sci. Total Environ..

[10] Zhang Z., Wang Y., Tan F., Bao M., Zhang L., Rodgers T.F., Chen J. (2020). Characteristics and risk assessment of organophosphorus flame retardants in urban road dust of Dalian, Northeast China. Sci. Total Environ..

[11] Tao F., Sellström U., De Wit C.A. (2019). Organohalogenated Flame Retardants and Organophosphate Esters in Office Air and Dust from Sweden. Environ. Sci. Technol..

[12] Li W.H., Shi Y.L., Gao L.H., Wu C.D., Liu J.M., Cai Y.Q. (2018). Occurrence, distribution and risk of organophosphate esters in urban road dust in Beijing, China. Environ. Pollut..

[13] Hou M.M., Shi Y.L., Na G.S., Zhao Z.S., Cai Y.Q. (2021). Increased Human Exposure to Organophosphate Esters via Ingestion of Drinking Water from Water Dispensers: Sources, Influencing Factors, and Exposure Assessment. Environ. Sci. Technol. Lett..

[14] Regnery J., Püttmann W. (2010). Seasonal fluctuations of organophosphate concentrations in precipitation and storm water runoff. Chemosphere.

[15] Han X., Hao Y.F., Li Y.M., Yang R.Q., Wang P., Zhang G.X., Zhang Q.H., Jiang G.B. (2020). Occurrence and distribution of organophosphate esters in the air and soils of Ny-Alesund and London Island, Svalbard, Arctic. Environ. Pollut..

[16] Yadav I.C., Devi N.L., Li J., Zhang G., Covaci A. (2018). Concentration and spatial distribution of organophosphate esters in the soil-sediment profile of Kathmandu Valley, Nepal: Implication for risk assessment. Sci. Total Environ..

[17] Li J., Tang J.H., Mi W.Y., Tian C.G., Emeis K.C., Ebinghaus R., Xie Z.Y. (2018). Spatial Distribution and Seasonal Variation of Organophosphate Esters in Air above the Bohai and Yellow Seas, China. Environ. Sci. Technol..

[18] Marklund A., Andersson B., Haglund P. (2003). Screening of organophosphorus compounds and their distribution in various indoor environments. Chemosphere.

[19] Lai S.C., Xie Z.Y., Song T.L., Tang J.H., Zhang Y.Y., Mi W.Y., Peng J.H., Zhao Y., Zou S.C., Ebinghaus R. (2015). Occurrence and dry deposition of organophosphate esters in atmospheric particles over the northern South China Sea. Chemosphere.

[20] Li J., Xie Z.Y., Mi E.Y., Lai S.C., Tian C.G., Emeis K.C., Ebinghaus R. (2017). Organophosphate Esters in Air, Snow, and Seawater in the North Atlantic and the Arctic. Environ. Sci. Technol..

[21] Huang Y.C., Tan H.L., Li L.Z., Yang L., Sun F.J., Li J., Gong X., Chen D. (2020). A broad range of organophosphate tri- and di-esters in house dust from Adelaide, South Australia: Concentrations, compositions, and human exposure risks. Environ. Int..

[22] Wang G.G., Liu Y., Zhao X.D., Tao W., Wang H.X. (2019). Geographical distributions and human exposure of organophosphate esters in college library dust from Chinese cities. Environ. Pollut..

[23] Smythe T.A., Mattioli L.C., Letcher R.J. (2020). Distribution behaviour in body compartments and in ovo transfer of flame retardants in North American Great Lakes herring gulls. Environ. Pollut..

[24] Hou M.M., Shi Y.L., Jin Q., Cai Y.Q. (2020). Organophosphate esters and their metabolites in paired human whole blood, serum, and urine as biomarkers of exposure. Environ. Int..

[25] Wang G.W., Shi H.H., Du Z.K., Chen H.Y., Peng J.B., Gao S.X. (2017). Bioaccumulation mechanism of organophosphate esters in adult zebrafish (*Danio rerio*). Environ. Pollut..

[26] Long S.X., Hamilton P.B., Fu B., Xu J., Han L.C., Suo X.H., Lai Y.Q., Shen G.F., Xu F.L., Li B.G. (2023). Bioaccumulation and emission of organophosphate esters in plants affecting the atmosphere’s phosphorus cycle. Environ. Int..

[27] Deng X., Yin H.L., HE W.L., Luo Y., Wu D., Luo L., Chen J. (2019). Distribution and migration of OPEs in soil profile and crops in urban and suburban areas of Chengdu. Environ. Chem..

[28] Picó Y., Campo J., Alfarhan A.H., El-Sheikh M.A., Barceló D. (2021). A reconnaissance study of pharmaceuticals, pesticides, perfluoroalkyl substances and organophosphorus flame retardants in the aquatic environment, wild plants and vegetables of two Saudi Arabia urban areas: Environmental and human health risk assessment. Sci. Total Environ..

[29] Mäkinen M.S.E., Mäkinen M.R.A., Koistinen J.T.B., Pasanen A.L., Pasanen P.O., Kalliokoski P.J., Korpi A.M. (2009). Respiratory and dermal exposure to organophosphorus flame retardants and tetrabromobisphenol A at five work environments. Environ. Sci. Technol..

[30] Meeker J.D., Cooper E.M., Stapleton H.M., Hauser R. (2013). Urinary metabolites of organophosphate flame retardants: Temporal variability and correlations with house dust concentrations. Environ. Health Perspect..

[31] Zheng G.M., Schreder E., Dempsey J.C., Uding N., Chu V., Andres G., Sathyanarayana S., Salamova A. (2021). Organophosphate Esters and Their Metabolites in Breast Milk from the United States: Breastfeeding Is an Important Exposure Pathway for Infants. Environ. Sci. Technol. Lett..

[32] Su G.Y., Crump D., Letcher R.J., Kennedy S.W. (2014). Rapid in vitro metabolism of the flame retardant triphenyl phosphate and effects on cytotoxicity and mRNA expression in chicken embryonic hepatocytes. Environ. Sci. Technol..

[33] Tran C.M., Lee H.J., Lee B.C., Ra J.S., Kim K.T. (2021). Effects of the chorion on the developmental toxicity of organophosphate esters in zebrafish embryos. J. Hazard. Mater..

[34] Sutha J., Anila P.A., Umamaheswari S., Ramesh M., Narayanasamy A., Poopal R.K., Ren Z.M. (2020). Biochemical responses of a freshwater fish *Cirrhinus mrigala* exposed to tris(2-chloroethyl) phosphate (TCEP). Environ. Sci. Pollut. Res..

[35] Hales B.F., Robaire B. (2020). Effects of brominated and organophosphate ester flame retardants on male reproduction. Andrology.

[36] Hou R., Xu Y.P., Wang Z.J. (2016). Review of OPFRs in animals and humans: Absorption, bioaccumulation, metabolism, and internal exposure research. Chemosphere.

[37] Qadeer A., Mubeen S., Liu M.Y., Bekele T.G., Ohoro C.R., Adeniji A.O., Alraih A.M., Ajmal Z., Alshammari A.S., Al-Hadeethi Y. (2024). Global environmental and toxicological impacts of polybrominated diphenyl ethers versus organophosphate esters: A comparative analysis and regrettable substitution dilemma. J. Hazard. Mater..

[38] Sun L.W., Tan H.N., Peng T., Wang S.S., Xu W.B., Qian H.F., Jin Y.X., Fu Z.W. (2016). Developmental neurotoxicity of organophosphate flame retardants in early life stages of Japanese medaka (*Oryzias latipes*). Environ. Toxicol. Chem..

[39] Hammel S.C., Nordone S., Zhang S., Lorenzo A.M., Eichner B., Moody M.A., Harrington L., Gandee J., Schmidt L., Smith S. (2022). Infants’ diminished response to DTaP vaccine is associated with exposure to organophosphate esters. Sci. Total Environ..

[40] Lee G., Kim S., Bastiaensen M., Malarvannan G., Poma G., Casero N.C., Gys C., Covaci A., Le S., Lim J.E. (2020). Exposure to organophosphate esters, phthalates, and alternative plasticizers in association with uterine fibroids. Environ. Res..

[41] Liu Y.H., Li Y., Dong S.S., Han L., Guo R.X., Fu Y.R., Zhang S.H., Chen J.Q. (2021). The risk and impact of organophosphate esters on the development of female-specific cancers: Comparative analysis of patients with benign and malignant tumors. J. Hazard. Mater..

[42] Yadav I.C., Devi N.L., Li J., Zhang G. (2018). Organophosphate ester flame retardants in Nepalese soil: Spatial distribution, source apportionment and air-soil exchange assessment. Chemosphere.

[43] Pang L., Liu J.F., Yin Y.G., Shen M.H. (2013). Evaluating the sorption of organophosphate esters to different sourced humic acids and its effects on the toxicity to Daphnia magna. Environ. Toxicol. Chem..

[44] Wang Q.Z., Zhao H.X., Bekele T.G., Qu B.C., Chen J.W. (2021). Organophosphate esters (OPEs) in wetland soil and Suaeda salsa from intertidal Laizhou Bay, North China: Levels, distribution, and soil-plant transfer model. Sci. Total Environ..

[45] You J., Chen Z.M., Hou X.Y., Guo J.S., Wang C.C., Gao J.M. (2022). Occurrence, potential sources and risks of organophosphate esters in the high-elevation region, Tibet, China. Sci. Total Environ..

[46] Liu M.Y., Guo C.S., Zhu C.F., Lv J.F., Yang W.L., Wu L.L., Xu J. (2022). Vertical profile and assessment of soil pollution from a typical coking plant by suspect screening and non-target screening using GC/QTOF-MS. Sci. Total Environ..

[47] Ge X., Ma S.T., Zhang X.L., Yang Y., Li G.Y., Yu Y.X. (2020). Halogenated and organophosphorus flame retardants in surface soils from an e-waste dismantling park and its surrounding area: Distributions, sources, and human health risks. Environ. Int..

[48] Yu Y.Y., Yu X.L., Zhang D.Q., Jin L., Huang J.H., Zhu X.F., Sun J.T., Yu M., Zhu L.Z. (2023). Biotransformation of Organophosphate Esters by Rice and Rhizosphere Microbiome: Multiple Metabolic Pathways, Mechanism, and Toxicity Assessment. Environ. Sci. Technol..

[49] Yu Y.Y., Huang J.H., Jin L., Yu M., Yu X.L., Zhu X.F., Sun J.T., Zhu L.Z. (2023). Translocation and metabolism of tricresyl phosphate in rice and microbiome system: Isomer-specific processes and overlooked metabolites. Environ. Int..

[50] Yu X.L., Li M., Tang S.Y., Wei Z., Yu Y.Y., Sun J.T., Lu G.N., Yin H. (2021). Photocatalysis of Tris-(2-chloroethyl) phosphate by ultraviolet driven peroxymonosulfate oxidation process: Removal performance, energy evaluation and toxicity on bacterial metabolism network. Chem. Eng. J..

[51] Yu X.L., Yin H., Peng H., Lu G.N., Liu Z.H., Li H.Y., Dang Z. (2020). Degradation mechanism, intermediates and toxicology assessment of tris-(2-chloroisopropyl) phosphate using ultraviolet activated hydrogen peroxide. Chemosphere.

[52] Moreno E.K.G., Garcia L.F., Lobón G.S., Brito L.B., Oliveira G.A.R., Luque R., Gil E.D.S. (2019). Ecotoxicological assessment and electrochemical remediation of doxorubicin. Ecotox. Environ. Safe..

[53] Sun J.T., Pan L.L., Tsang D.C.W., Li Z.H., Zhu L.Z., Li X.D. (2018). Phthalate esters and organochlorine pesticides in agricultural soils and vegetables from fast-growing regions: A case study from eastern China. Environ. Sci. Pollut. Res..

[54] Sun J.T., Pan L.L., Zhan Y., Lu H.N., Tsang D.C.W., Liu W.X., Wang X.L., Li X.D., Zhu L.Z. (2016). Contamination of phthalate esters, organochlorine pesticides and polybrominated diphenyl ethers in agricultural soils from the Yangtze River Delta of China. Sci. Total Environ..

[55] Yang Q.W., Mei X.X., Sun J.X., Hu Y. (2018). Sources, Distribution and Major Transformation Process of Typical Endocrine Disruptors in the Environment. Asian J. Ecotoxicol..

[56] US Environmental Protection Agency (2011). USEPA Exposure Factors Handbook.

[57] Zhang X.L., Zou W., Mu L., Chen Y.M., Ren C.X., Hu X.G., Zhou Q.X. (2016). Rice ingestion is a major pathway for human exposure to organophosphate flame retardants (OPFRs) in China. J. Hazard. Mater..

[58] Liu C.Y., Wang L.M., Gao X.X., Huang Z.J., Zhang X., Zhao Z.P., Li C., Zhang M. (2022). Intake of vegetables and fruit among adults in China in 2018. Chin. J. Prev. Contr. Chron. Dis..

[59] Li L., Ouyang Y.F., Wang H.J., Huang F.F., Wang Y., Zhang J.G., Sui C., Du W.W., Jia X.F., Jiang H.R. (2020). Status of fruit and vegetable intake among children and adolescents in 15 provinces of China. Chin. J. Health Educ..

[60] Kurt-Karakus P., Alegria H., Birgul A., Gungormus E., Jantunen L. (2018). Organophosphate ester (OPEs) flame retardants and plasticizers in air and soil from a highly industrialized city in Turkey. Sci. Total Environ..

[61] Wang Y., Li Z.Y., Tan F., Xu Y., Zhao H.X., Chen J.W. (2020). Occurrence and air-soil exchange of organophosphate flame retardants in the air and soil of Dalian, China. Environ. Pollut..

[62] Tang J.F., Sun J., Ke Z.Y., Yin H.L., Yang L., Yen H., Li X.H., Xu Y.Y. (2021). Organophosphate esters in surface soils from a heavily urbanized region of Eastern China: Occurrence, distribution, and ecological risk assessment. Environ. Pollut..

[63] Cui K.Y., Wen J.X., Zeng F., Li S.C., Zhou X., Zeng Z.X. (2017). Occurrence and distribution of organophosphate esters in urban soils of the subtropical city, Guangzhou, China. Chemosphere.

[64] He J., Wang Z.X., Zhao L.Y., Ma H.B., Huang J., Li H.Y., Mao X.X., Huang T., Gao H., Ma J.M. (2021). Gridded emission inventory of organophosphorus flame retardants in China and inventory validation. Environ. Pollut..

[65] He M.J., Yang T., Yang Z.H., Zhou H., Wei S.Q. (2018). Current State, Distribution, and Sources of Phthalate Esters and Organophosphate Esters in Soils of the Three Gorges Reservoir Region, China. Arch. Environ. Contam. Toxicol..

[66] Li X.H., Ma J., Fang D., Shi T.R., Gong Y.W. (2020). Organophosphate Flame Retardants in Soils of Zhejiang Province, China: Levels, Distribution, Sources, and Exposure Risks. Arch. Environ. Contam. Toxicol..

[67] Sun Y.L., Zhu H.K. (2021). A pilot study of organophosphate esters in surface soils collected from Jinan City, China: Implications for risk assessments. Environ. Sci. Pollut. Res..

[68] Zhang W.W., Wang P., Li Y.M., Wang D., Matsiko J., Yang R.Q., Sun H.Z., Hao Y.F., Zhang Q.H., Jiang G.B. (2019). Spatial and temporal distribution of organophosphate esters in the atmosphere of the Beijing-Tianjin-Hebei region, China. Environ. Pollut..

[69] Ji Y., Wang Y., Yao Y.M., Ren C., Lan Z.H., Fang X.G., Zhang K., Sun W.J., Alder A.C., Sun H.W. (2019). Occurrence of organophosphate flame retardants in farmland soils from northern China: Primary source analysis and risk assessment. Environ. Pollut..

[70] Mihajlović I., Miloradov M.V., Fries E. (2011). Application of Twisselmann Extraction, SPME, and GCMS To Assess Input Sources for Organophosphate Esters into Soil. Environ. Sci. Technol..

[71] Casas G., Martinez-Varela A., Vila-Costa M., Jiménez B., Dachs J. (2021). Rain amplification of persistent organic pollutants. Environ. Sci. Technol..

[72] He J.H., Li J.F., Ma L.Y., Wu N., Zhang Y., Niu Z.G. (2019). Large-scale distribution of organophosphate esters (flame retardants and plasticizers) in soil from residential area across China: Implications for current level. Sci. Total Environ..

[73] Xu L., Hu Q.P., Liu J., Liu S.H., Liu C.J., Deng Q.Y., Zeng X.Y., Yu Z.Q. (2019). Occurrence of organophosphate esters and their diesters degradation products in industrial wastewater treatment plants in China: Implication for the usage and potential degradation during production processing. Environ. Pollut..

[74] Gao L.H., Shi Y.L., Li W.H., Liu J.M., Cai Y.Q. (2016). Occurrence and distribution of organophosphate triesters and diesters in sludge from sewage treatment plants of Beijing, China. Sci. Total Environ..

[75] Fu L.F., Du B.B., Wang F., Lam J.C.W., Zeng L.X., Zeng E.Y. (2017). Organophosphate triesters and diester degradation products in municipal sludge from wastewater treatment plants in China: Spatial patterns and ecological implications. Environ. Sci. Technol..

[76] Gong X.Y., Zhang W.J., Zhang S.Y., Wang Y., Zhang X.Y., Lu Y., Sun H.W., Wang L. (2021). Organophosphite antioxidants in mulch films are important sources of organophosphate pollutants in farmlands. Environ. Sci. Technol..

[77] Veen I.V.D., Boer J.D. (2012). Phosphorus flame retardants: Properties, production, environmental occurrence, toxicity and analysis. Chemosphere.

[78] Zhang Q., Wang Y.X., Jiang X.X., Xu H.Z., Luo Y.Q., Long T.T., Li J., Xing L.Q. (2021). Spatial occurrence and composition profile of organophosphate esters (OPEs) in farmland soils from different regions of China: Implications for human exposure. Environ. Pollut..

[79] Wang Y., Yao Y.M., Li W.H., Zhu H.K., Wang L., Sun H.W., Kannan K. (2019). A nationwide survey of 19 organophosphate esters in soils from China: Spatial distribution and hazard assessment. Sci. Total Environ..

[80] Sang Y.Z., Li W.H., Liu J.M., Qu C., Zhao Y.J., Cai H.M., Hong S.C. (2020). Simultaneous Determination of 13 Organophosphate Esters in Greenhouse Soil by Gas Chromatography-Mass Spectrometry. J. Instrum. Anal..

[81] Fisk P.R., Girling A.E., Wildey R.J. (2003). Prioritisation of Flame Retardants for Environmental Risk Assessment.

[82] Regnery J., Püttmann W. (2010). Occurrence and fate of organophosphorus flame retardants and plasticizers in urban and remote surface waters in Germany. Water Res..

[83] Kawagoshi Y., Nakamura S., Fukunaga I. (2002). Degradation of organophosphoric esters in leachate from a sea-based solid waste disposal site. Chemosphere.

[84] Chen Y.Q., Zhang Q., Luo T.W., Xing L.Q., Xu H.Z. (2019). Occurrence, distribution and health risk assessment of organophosphate esters in outdoor dust in Nanjing, China: Urban vs. rural areas. Chemosphere.

[85] Kim U.J., Kannan K. (2018). Occurrence and distribution of organophosphate flame retardants/plasticizers in surface waters, tap water, and rainwater: Implications for human exposure. Environ. Sci. Technol..

[86] Shi Y.L., Gao L.H., Li W.H., Wang Y., Liu J.M., Cai Y.Q. (2016). Occurrence, distribution and seasonal variation of organophosphate flame retardants and plasticizers in urban surface water in Beijing, China. Environ. Pollut..

[87] Xing L.Q., Tao M., Zhang Q., Kong M., Sun J., Jia S.Y., Liu C.H. (2020). Occurrence, spatial distribution and risk assessment of organophosphate esters in surface water from the lower Yangtze River Basin. Sci. Total Environ..

[88] Zeng F., Cui K.Y., Xie Z.Y., Wu L.N., Luo D.L., Chen L.X., Lin Y.J., Liu M., Sun G.X. (2009). Distribution of phthalate esters in urban soils of subtropical city, Guangzhou, China. J. Hazard. Mater..

[89] Wang X.T., Wang X.K., Zhang Y., Chen L., Sun Y.F., Li M., Wu M.H. (2014). Short-and medium-chain chlorinated paraffins in urban soils of Shanghai: Spatial distribution, homologue group patterns and ecological risk assessment. Sci. Total Environ..

[90] Huang Y.M., Chen L.G., Feng Y.B., Ye Z.X., He Q.S., Feng Q.H., Qing X., Liu M., Gao B. (2016). Short-chain chlorinated paraffins in the soils of two different Chinese cities: Occurrence, homologue patterns and vertical migration. Sci. Total Environ..

[91] Luo Q., Shan Y., Muhammad A., Wang S.y., Sun L.N., Wang H. (2018). Levels, distribution, and sources of organophosphate flame retardants and plasticizers in urban soils of Shenyang. China. Environ. Sci. Pollut. Res..

[92] He C., Wang X.Y., Tang S.Y., Thai P., Li Z.R., Baduel C., Mueller J.F. (2018). Concentrations of Organophosphate Esters and Their Specific Metabolites in Food in Southeast Queensland, Australia: Is Dietary Exposure an Important Pathway of Organophosphate Esters and Their Metabolites?. Environ. Sci. Technol..

[93] Poma G., Sales C., Bruyland B., Christia C., Goscinny S., Loco J.V., Covaci A. (2018). Occurrence of Organophosphorus Flame Retardants and Plasticizers (PFRs) in Belgian Foodstuffs and Estimation of the Dietary Exposure of the Adult Population. Environ. Sci. Technol..

[94] Poma G., Glynn A., Malarvannan G., Covaci A., Darnerud P.O. (2017). Dietary intake of phosphorus flame retardants (PFRs) using Swedish food market basket estimations. Food Chem. Toxicol..

[95] Tian Y.X. (2023). Study on the Occurrence Status and Environmental Risk of Organophosphate Esters in Soils of Beijing Urban Parks.

[96] Wang S.Y. (2018). Pollution Characteristics and Risk Assessment of Organic Phosphate Easters of the Liaohekou.

[97] Schmidt N., Castro-Jiménez J., Oursel B., Sempéré R. (2021). Phthalates and organophosphate esters in surface water, sediments and zooplankton of the NW Mediterranean Sea: Exploring links with microplastic abundance and accumulation in the marine food web. Environ. Pollut..

[98] Xing L.Q., Zhang Y.Y., Chang S., Tao L.Y., Su G.Y. (2023). Uptake, accumulation and translocation of traditional and novel organophosphate esters by rice seedlings in the presence of micro(nano)-polystyrene plastics: Effects of concentration and size of particles. Sci. Total Environ..

[99] European Commission (2017). Identification and Evaluation of Data On flame Retardants in Consumer Products. https://www.europeanfiresafetyalliance.org/publications/identification-and-evaluation-of-data-on-flame-retardants-in-consumer-products/.

[100] (2023). USEPA Mid Atlantic Risk Assessment, Regional Screening Levels (RSLs)-Generic Tables. http://www.epa.gov/region9/superfund/prg.

[101] Ding J.J., Shen X.L., Liu W.P., Covaci A., Yang F.X. (2015). Occurrence and risk assessment of organophosphate esters in drinking water from Eastern China. Sci. Total Environ..

[102] Ali N., Dirtu A.C., Eede N.V.D., Goosey E., Harrad S., Neels H., Mannetje A., Coakley J., Douwes J., Covaci A. (2012). Occurrence of alternative flame retardants in indoor dust from New Zealand: Indoor sources and human exposure assessment. Chemosphere.

[103] USDoE (2011). The Risk Assessment Information System (RAIS).

[104] MEPC, Ministry of Environmental Protection of the People’s Republic of China (2013). Exposure Factors Handbook of Chinese Population.

[105] USEPA (US Environmental Protection Agency) (2001). Supplemental Guidance for Developing Soil Screening Levels for Superfund Sites.

