# Supplementary material for: Distribution and Risk Assessment of Organophosphate Esters in Agricultural Soils and Plants in the Coastal Areas of South China
Source: Toxics. 2024 Apr 12;12(4):286. doi: 10.3390/toxics12040286 (PMC11054690; doi:10.3390/toxics12040286)
Supplement: Supplementary file 1 [file toxics-12-00286-s001.zip › toxics-2945155-supplementary.pdf]

# Supporting Information

# Distribution and Risk Assessment of Organophosphate Esters in Agricultural Soils and Plants in the Coastal Areas of South China

**Wangxing Luo [1,2], Siyu Yao [3], Jiahui Huang [1,*], Haochuan Wu [4], Haijun Zhou [1], Mingjiang Du [1], Ling Jin [3] and Jianteng Sun [1,*]**

[1] School of Environmental Science and Engineering, Guangdong University of Petrochemical Technology, Maoming 525000, China; liubei890118@163.com (W.L.); zhouhaijun_2000@126.com (H.Z.); 15818969010@163.com (M.D.)
[2] Iron Man Environmental Technology Co., Ltd., Foshan 528000, China
[3] Department of Civil and Environmental Engineering, The Hong Kong Polytechnic University, Hung Hom, Kowloon 999077, Hong Kong; yaosy19@lzu.edu.cn (S.Y.); ling.jin@polyu.edu.hk (L.J.)
[4] School of Housing, Building and Planning, Universiti Sains Malaysia, George Town, Pulau Pinang 11800, Malaysia; wuhaochuan2024@163.com
* Correspondence: hjh960409@163.com (J.H.); sunjianteng@zju.edu.cn (J.S.)

**Text S1 The method of soil sample extraction and detection of TOC and pH in soil.**

**Plants:** After adding 20 mL of the extractant mixture to the glass centrifuge tube with the plant sample, shaken well. The sample was then placed in the ultrasonic extraction system for 20 min, followed by centrifugation at 4000 rpm for 10 min. After centrifugation, the supernatant was transferred to the chicken heart bottle. The ultrasonic extraction and centrifugation operation was repeated three times, and three supernatants were combined into the chicken heart bottle. The supernatant in the flask was evaporated to approximately 2 mL using nitrogen and then left to purify. A glass column packed with 4 cm of neutral silica gel, and 1 cm of anhydrous sodium sulfate, was used for sample purification. Activated the column with 10 mL of n-hexane and discarded it. Connected the commercialized Graphitized carbon black (GCB) column (activated with 6 mL of acetone) to the bottom of the silica gel column, loaded the sample of nitrogen-blown, and washed the combined column with 20 mL of acetone. When the rinsing solution was about to flow out, replaced the waste liquid bottle with a chicken heart bottle. Blew the solution in the chicken heart bottle to near dryness. The volume was then adjusted by adding 1 mL hexane to the flask and shaking well, which was sealed and stored for detection.

**Soil:** Weighed 1 g soil sample and placed into a 30 mL glass centrifuge tube. The extractant was a hexane: dichloromethane: acetone (2:2:1; v/v) mixture. Subsequently, 10 mL of the extractant mixture was added to the glass centrifuge tube with the soil sample, and shaken well. The sample was placed in the ultrasonic extraction system: the time was set to 20 min. The centrifugation setting was 2000 r/min, 10 min. The supernatant was transferred to the chicken heart bottle at the end of centrifugation. The above ultrasonic extraction and centrifugation operation was repeated three times, and three supernatants were combined into the chicken heart bottle. The flask containing the supernatant was nitrogen blown to near dryness, and the volume was fixed by adding hexane to the flask to a total volume of 1 mL, and shaken well, which was sealed and stored at 4°C for detection.

**TOC and pH:** Soil pH was measured using a pH meter (Inesa, Shanghai, China) with a soil/water ratio of 1:2.5. Total organic carbon (TOC) was measured using an Elementar Vavio EL III elemental analyzer (Hanau, Germany).

**Table S1. Details of the 37 sampling sites.**

| Sampling location | East Longitude | Northern Latitude |
|---|---|---|
| CZX | 117.100806 | 23.572944 |
| FCGS | 108.515856 | 21.599694 |
| FCGL | 108.212319 | 21.506575 |
| MMT | 110.951478 | 21.563847 |
| MMN | 111.049783 | 21.463131 |
| MMJ | 111.262819 | 21.423122 |
| MML | 111.419615 | 21.540649 |
| MMY | 111.184644 | 21.500889 |
| QZD | 118.979964 | 25.001342 |
| QZM | 118.950714 | 25.158578 |
| QZB | 118.760797 | 24.766167 |

| | | |
|---|---|---|
| XMZ | 118.020906 | 24.450444 |
| XML | 118.193719 | 24.574706 |
| YJS | 111.681011 | 21.595306 |
| YJL | 111.822364 | 21.713825 |
| ZJT | 110.409367 | 21.004358 |
| ZJB | 110.375092 | 21.169489 |
| ZJJ | 110.897219 | 21.402325 |
| ZZT | 117.270911 | 23.635269 |
| ZZB | 117.610719 | 23.795119 |
| ZZS | 117.894083 | 24.079042 |
| ZZZ | 118.097258 | 24.257917 |
| SWN | 115.420097 | 22.694058 |
| BHX | 109.712433 | 21.481092 |
| STB | 116.780892 | 23.367864 |
| JYD | 116.439492 | 22.949333 |
| GXQZZ | 108.734553 | 21.637219 |
| DGF | 113.577308 | 22.88475 |
| JMC | 113.010436 | 21.981544 |
| ZHG | 113.237053 | 21.922653 |
| ZSL | 113.550114 | 22.491089 |
| HZX | 114.66896 | 22.77592 |
| GZX | 113.566014 | 22.718153 |
| GZT | 113.496656 | 22.942033 |
| GZN | 113.504078 | 23.07355 |
| SZM | 113.771397 | 22.741106 |
| SZX | 114.327478 | 22.607647 |

Note: Guangdong Province included Chaozhou (CZ), Maoming (MM), Yangjiang (YJ), Zhanjiang (ZJ), Shanwei (SW), Shantou (ST), Jieyang (JY), Dongguan (DG), Jiangmen (JM), Zhuhai (ZH), Zhongshan (ZS), Huizhou (HZ), Guangzhou (GZ), Shenzhen (SZ); Guangxi Zhuang Autonomous Region included Fangchenggang (FCG), Beihai (BH), and Qinzhou (GXQZ); and Fujian Province included Quanzhou (QZ), Xiamen (XM), and Zhangzhou (ZZ).

**Table S2 GS-MS/MS parameter of OPEs**

| Acronym | CAS No. | Chemical formula | Mol. Wt. | Quantifier/Qualifier |
|---|---|---|---|---|
| TCEP | 115-96-8 | C6H12Cl3O4P | 285.5 | 249/143 |
| TDCIPP | 13674-87-8 | C9H15Cl6O4P | 430.9 | 191/381 |
| TPHP | 115-86-6 | C18H15O4P | 326.3 | 170/228 |
| EHDPHP | 1241-94-7 | C20H27O4P | 362.4 | 251/170 |
| TCPP | 6145-73-9 | C9H18Cl3O4P | 327.5 | 125/99 |
| ToCP | 78-30-8 | C21H21O4P | 368.37 | 179/165 |
| TpCP | 78-32-0 | C21H21O4P | 368.37 | 165/243 |
| TmCP | 563-04-2 | C21H21O4P | 368.37 | 165/198 |

**Table S3. Parameters for calculating PNECsoil, RfD, and SFO of OPEs in soils**

| OPEs | PNECsoil(ng/g) | RfD (mg/kg/d) | SFO $(mg/kg/d)^{-1}$ |
| --- | --- | --- | --- |
| TCEP | 386[1,2] | 7.00E-03[3,4,5] | 2.00E-02[3,4,5] |
| TDCIPP | 320[1,2] | 2.00E-02[3,4,5] | 3.10E-02[3,4,5] |
| TPHP | 130[1,2] | 7.00E-02[3,4,5] | - |
| EHDPP | 30.2[1,2] | - | - |
| TcPP | - | - | - |
| ToCP | - | - | - |
| TpCP | - | - | - |
| TmCP | - | - | - |

Note: PNECsoil is the expected concentration of ineffective stress in soil (ng/g dry weight). RfD is the corresponding oral reference dose, mg/kg-day; SFO is the corresponding oral cancer slope factor $(mg/kg\text{-}day)^{-1}$.

**Table S4 Calculation parameters and values used in the health risk assessment model to evaluate exposure risks of OPEs in soils**

| parameter | units | Exposure | | References |
| --- | --- | --- | --- | --- |
| | | Children | Adult | |
| IR | mg/day | 50 | 20 | [3,6-9] |
| EF | day/year | 350 | 350 | [3,6-9] |
| ED | year | 6 | 24 | [3,6-9] |
| BW | kg | 29 | 63 | [3,6-9] |
| AT | Day | 2190 (non-carcinogenic)<br>25550 (carcinogenic) | 8760 (non-carcinogenic)<br>25550 (carcinogenic) | [3,6-9] |
| HR | $m^3$/day | 7.6 | 16 | [3,6-9] |
| PEF | $m^3$/kg | $1.4 \times 10^9$ | $1.4 \times 10^9$ | [3,6-9] |
| SA | $cm^2$ | 2800 | 5700 | [3,6-9] |
| AF | mg/$cm^2$ | 0.2 | 0.07 | [3,6-9] |
| ABS | unitless | 0.1 | 0.1 | [3,6-9] |
| GIABS | unitless | 1 | 1 | [3,6-9] |

Note: CDIingest, CDIdermal, and CDIinhale represent the chronic daily intake through feeding, skin contact, and inhalation pathways, respectively, (mg/kg-day); Csoil is the concentration of OPEs in the soil, mg/kg; IR is the soil ingestion rate, mg/day; EF is the exposure frequency, day/year; ED is the exposure cycle, year; ET is the daily exposure time, h/day; BW is the weight, kg; AT is the average exposure time, day; HR is the air intake rate, $m^3$/day; PEF is the particulate matter emission factor; SA is the surface area of skin exposure,$cm^2$; AF is a relative skin adhesion factor, mg/$cm^2$; ABS is a skin absorption factor, no units; CF is the conversion coefficient,$10^{-6}$ kg/mg. GIABS is a gastrointestinal absorption factor, without units; X represents the number of pollutants.

**Table S5 Daily consumption (g/day) and body weight (kg) for general Chinese Children and Adult.**

| parameter | Children | Adult | References |
|---|---|---|---|
| DC | 166.2 | 369.1 | [10,11] |
| BW | 29 | 63 | [3,6-9] |

Note: EDI: estimate of dietary intake, (ng/kg bw/day), C represents the concentration of OPE in crops (ng/g dry weight), DC is the daily crop consumption of consumers (g/day), BW is the body weight (kg).

**Table S6 Estimated Risk quotient (RQ) of different OPEs for soil and total RQ in the coastal areas of South China**

| Sampling location | TCEP | TDCIPP | TPHP | EHDPP | ∑OPE |
|---|---|---|---|---|---|
| CZX | 0.238979591 | 0.696541055 | 0.071840227 | 0.152696009 | 1.160056882 |
| FCGS | 0.034016473 | 0.311417902 | 0.055894602 | 0.162166121 | 0.563495098 |
| FCGL | 0.04241979 | 0.457208664 | 0.074160865 | 0.151836685 | 0.725626003 |
| MMT | 0.045015115 | 0.526625109 | 0.06162262 | 0.115211623 | 0.748474467 |
| MMN | 0.033005071 | 0.260999567 | 0.107911532 | 0.128104415 | 0.530020586 |
| MMJ | 0.025830989 | 0.094764697 | 0.068885685 | 0.122152676 | 0.311634047 |
| MML | 0.04337308 | 0.482391025 | 0.126702868 | 0 | 0.652466973 |
| MMY | 0.029072546 | 0.157715764 | 0 | 0 | 0.186788311 |
| QZD | 0.042208216 | 0.250793137 | 0.058780735 | 0.134012379 | 0.485794467 |
| QZM | 0.079026512 | 0.757063827 | 0.055607215 | 0.127022782 | 1.018720336 |
| QZB | 0.058052632 | 0.223829256 | 0.075237199 | 0 | 0.357119087 |
| XMZ | 0.062151634 | 0.617254481 | 0.05285294 | 0 | 0.732259055 |
| XML | 0.052081659 | 0.768307409 | 0.057621669 | 0.122627016 | 1.000637753 |
| YJS | 0.02594447 | 0.050534561 | 0.058211131 | 0 | 0.134690163 |
| YJL | 0.072481089 | 0.721633021 | 0.053004272 | 0.122099263 | 0.969217645 |
| ZJT | 0.033921667 | 0.384479425 | 0.060741003 | 0.189596043 | 0.668738138 |
| ZJB | 0.032419602 | 0.520466585 | 0.074047523 | 0.186648602 | 0.813582312 |
| ZJJ | 0.01306218 | 0.226912028 | 0.079349715 | 0.233744647 | 0.55306857 |
| ZZT | 0.049701595 | 0.566353476 | 0.063685837 | 0.162874788 | 0.842615696 |
| ZZB | 0.098285002 | 0.43310989 | 0.05298945 | 0.147183893 | 0.731568235 |
| ZZS | 0.291244005 | 0.484868555 | 0.065080927 | 0.147944461 | 0.989137948 |
| ZZZ | 0.018322122 | 0.10761492 | 0.030453195 | 0 | 0.156390236 |
| SWN | 0.049993993 | 0.62243652 | 0.061073678 | 0.172469768 | 0.905973958 |
| BHX | 0.037526812 | 0.091573897 | 0.051207541 | 0.159948511 | 0.340256762 |
| STB | 0.030048255 | 0.37502394 | 0.063184005 | 0.787949622 | 1.256205822 |
| JYD | 0.046094393 | 0.602602872 | 0.047446706 | 0.698681097 | 1.394825069 |
| GXQZZ | 0.033610868 | 0.521385379 | 0.051290728 | 0.127609974 | 0.733896949 |
| DGF | 0.010325102 | 0.09312168 | 0.050348701 | 0.117017434 | 0.270812917 |
| JMC | 0.038511831 | 0.38193769 | 0.109014428 | 0.136260418 | 0.665724368 |
| ZHG | 0.401065352 | 0.49172085 | 0.034365433 | 0.114181264 | 1.0413329 |

| | | | | | |
|---|---|---|---|---|---|
| ZSL | 0.28762569 | 0.724661056 | 0.060675431 | 0.133634573 | 1.20659675 |
| HZX | 0.085115137 | 0.523604179 | 0.03575378 | 0.114993698 | 0.759466793 |
| GZX | 0.0132741 | 0.332206666 | 0.050532428 | 2.38135931 | 2.777372503 |
| GZT | 0.032784548 | 0.37618099 | 0.044400656 | 0 | 0.453366194 |
| GZN | 0.089663043 | 0.61059038 | 0.056671592 | 0.231086583 | 0.988011598 |
| SZM | 0.069874735 | 0.621506117 | 0.105902912 | 0.134141741 | 0.931425505 |
| SZX | 0.072259081 | 0.627589636 | 0.03781831 | 0 | 0.737667027 |
| **maximum** | 0.401065352 | 0.768307409 | 0.126702868 | 2.38135931 | 2.777372503 |
| **minimum** | 0.010325102 | 0.050534561 | 0 | 0 | 0.134690163 |
| **mean** | 0.073469945 | 0.435054762 | 0.061199123 | 0.208520416 | 0.778244247 |

**Table S7 The hazard quotient (HQ) for different OPEs with two pathways in soils.**

**A: The hazard quotient (HQ) of ΣOPE with two pathways in soils**

| **Sampling location** | **children** | | | **adult** | | |
|---|---|---|---|---|---|---|
| | ingestion | Dermal contact | Total HQ | ingestion | Dermal contact | Total HQ |
| CZX | 4.04E-02 | 4.53E-02 | 8.57E-02 | 7.44E-03 | 1.49E-02 | 2.23E-02 |
| FCGS | 1.15E-02 | 1.29E-02 | 2.44E-02 | 2.12E-03 | 4.23E-03 | 6.35E-03 |
| FCGL | 1.62E-02 | 1.81E-02 | 3.43E-02 | 2.98E-03 | 5.95E-03 | 8.93E-03 |
| MMT | 1.82E-02 | 2.04E-02 | 3.86E-02 | 3.36E-03 | 6.69E-03 | 1.00E-02 |
| MMN | 1.02E-02 | 1.15E-02 | 2.17E-02 | 1.89E-03 | 3.76E-03 | 5.65E-03 |
| MMJ | 5.07E-03 | 5.68E-03 | 1.08E-02 | 9.34E-04 | 1.86E-03 | 2.80E-03 |
| MML | 1.71E-02 | 1.92E-02 | 3.63E-02 | 3.15E-03 | 6.28E-03 | 9.43E-03 |
| MMY | 6.82E-03 | 7.64E-03 | 1.45E-02 | 1.26E-03 | 2.51E-03 | 3.76E-03 |
| QZD | 1.07E-02 | 1.19E-02 | 2.26E-02 | 1.96E-03 | 3.92E-03 | 5.88E-03 |
| QZM | 2.74E-02 | 3.07E-02 | 5.81E-02 | 5.05E-03 | 1.01E-02 | 1.51E-02 |
| QZB | 1.14E-02 | 1.28E-02 | 2.43E-02 | 2.11E-03 | 4.20E-03 | 6.31E-03 |
| XMZ | 2.22E-02 | 2.48E-02 | 4.70E-02 | 4.08E-03 | 8.14E-03 | 1.22E-02 |
| XML | 2.52E-02 | 2.83E-02 | 5.35E-02 | 4.65E-03 | 9.27E-03 | 1.39E-02 |
| YJS | 3.88E-03 | 4.35E-03 | 8.23E-03 | 7.15E-04 | 1.43E-03 | 2.14E-03 |
| YJL | 2.59E-02 | 2.90E-02 | 5.48E-02 | 4.76E-03 | 9.50E-03 | 1.43E-02 |
| ZJT | 1.34E-02 | 1.51E-02 | 2.85E-02 | 2.48E-03 | 4.94E-03 | 7.42E-03 |
| ZJB | 1.70E-02 | 1.90E-02 | 3.59E-02 | 3.12E-03 | 6.23E-03 | 9.35E-03 |
| ZJJ | 7.44E-03 | 8.33E-03 | 1.58E-02 | 1.37E-03 | 2.73E-03 | 4.10E-03 |
| ZZT | 1.97E-02 | 2.21E-02 | 4.18E-02 | 3.63E-03 | 7.24E-03 | 1.09E-02 |
| ZZB | 2.06E-02 | 2.30E-02 | 4.36E-02 | 3.79E-03 | 7.56E-03 | 1.13E-02 |
| ZZS | 3.96E-02 | 4.43E-02 | 8.39E-02 | 7.29E-03 | 1.45E-02 | 2.18E-02 |
| ZZZ | 4.61E-03 | 5.16E-03 | 9.77E-03 | 8.49E-04 | 1.69E-03 | 2.54E-03 |
| SWN | 2.12E-02 | 2.38E-02 | 4.50E-02 | 3.91E-03 | 7.79E-03 | 1.17E-02 |
| BHX | 6.00E-03 | 6.72E-03 | 1.27E-02 | 1.10E-03 | 2.20E-03 | 3.31E-03 |
| STB | 1.29E-02 | 1.44E-02 | 2.72E-02 | 2.37E-03 | 4.72E-03 | 7.09E-03 |
| JYD | 2.03E-02 | 2.27E-02 | 4.30E-02 | 3.74E-03 | 7.45E-03 | 1.12E-02 |
| GXQZZ | 1.70E-02 | 1.91E-02 | 3.61E-02 | 3.13E-03 | 6.25E-03 | 9.38E-03 |

| | | | | | | |
|---|---|---|---|---|---|---|
| DGF | 3.56E-03 | 3.99E-03 | 7.55E-03 | 6.55E-04 | 1.31E-03 | 1.96E-03 |
| JMC | 1.39E-02 | 1.56E-02 | 2.96E-02 | 2.57E-03 | 5.12E-03 | 7.69E-03 |
| ZHG | 4.97E-02 | 5.56E-02 | 1.05E-01 | 9.15E-03 | 1.82E-02 | 2.74E-02 |
| ZSL | 4.56E-02 | 5.10E-02 | 9.66E-02 | 8.39E-03 | 1.67E-02 | 2.51E-02 |
| HZX | 2.17E-02 | 2.43E-02 | 4.60E-02 | 4.00E-03 | 7.98E-03 | 1.20E-02 |
| GZX | 1.02E-02 | 1.14E-02 | 2.15E-02 | 1.87E-03 | 3.73E-03 | 5.60E-03 |
| GZT | 1.31E-02 | 1.46E-02 | 2.77E-02 | 2.41E-03 | 4.80E-03 | 7.21E-03 |
| GZN | 2.45E-02 | 2.74E-02 | 5.19E-02 | 4.51E-03 | 9.00E-03 | 1.35E-02 |
| SZM | 2.31E-02 | 2.59E-02 | 4.90E-02 | 4.26E-03 | 8.50E-03 | 1.28E-02 |
| SZX | 2.33E-02 | 2.61E-02 | 4.94E-02 | 4.29E-03 | 8.56E-03 | 1.29E-02 |
| **maximum** | 4.97E-02 | 5.56E-02 | 1.05E-01 | 9.15E-03 | 1.82E-02 | 2.74E-02 |
| **minimum** | 3.56E-03 | 3.99E-03 | 7.55E-03 | 6.55E-04 | 1.31E-03 | 1.96E-03 |
| **mean** | 1.84E-02 | 2.06E-02 | 3.90E-02 | 3.39E-03 | 6.76E-03 | 1.01E-02 |

**B: The hazard quotient (HQ) of TCEP with two pathways in soils**

| **Sampling location** | **children** | | | **adult** | | |
|---|---|---|---|---|---|---|
| | ingestion | Dermal contact | Total HQ | ingestion | Dermal contact | Total HQ |
| CZX | 2.18E-02 | 2.44E-02 | 4.62E-02 | 4.01E-03 | 8.00E-03 | 1.20E-02 |
| FCGS | 3.10E-03 | 3.47E-03 | 6.57E-03 | 5.71E-04 | 1.14E-03 | 1.71E-03 |
| FCGL | 3.87E-03 | 4.33E-03 | 8.20E-03 | 7.12E-04 | 1.42E-03 | 2.13E-03 |
| MMT | 4.10E-03 | 4.60E-03 | 8.70E-03 | 7.56E-04 | 1.51E-03 | 2.26E-03 |
| MMN | 3.01E-03 | 3.37E-03 | 6.38E-03 | 5.54E-04 | 1.11E-03 | 1.66E-03 |
| MMJ | 2.35E-03 | 2.64E-03 | 4.99E-03 | 4.34E-04 | 8.65E-04 | 1.30E-03 |
| MML | 3.95E-03 | 4.43E-03 | 8.38E-03 | 7.28E-04 | 1.45E-03 | 2.18E-03 |
| MMY | 2.65E-03 | 2.97E-03 | 5.62E-03 | 4.88E-04 | 9.74E-04 | 1.46E-03 |
| QZD | 3.85E-03 | 4.31E-03 | 8.16E-03 | 7.09E-04 | 1.41E-03 | 2.12E-03 |
| QZM | 7.20E-03 | 8.07E-03 | 1.53E-02 | 1.33E-03 | 2.65E-03 | 3.97E-03 |
| QZB | 5.29E-03 | 5.93E-03 | 1.12E-02 | 9.74E-04 | 1.94E-03 | 2.92E-03 |
| XMZ | 5.67E-03 | 6.35E-03 | 1.20E-02 | 1.04E-03 | 2.08E-03 | 3.12E-03 |
| XML | 4.75E-03 | 5.32E-03 | 1.01E-02 | 8.74E-04 | 1.74E-03 | 2.62E-03 |
| YJS | 2.37E-03 | 2.65E-03 | 5.01E-03 | 4.36E-04 | 8.69E-04 | 1.30E-03 |
| YJL | 6.61E-03 | 7.40E-03 | 1.40E-02 | 1.22E-03 | 2.43E-03 | 3.64E-03 |
| ZJT | 3.09E-03 | 3.46E-03 | 6.56E-03 | 5.69E-04 | 1.14E-03 | 1.71E-03 |
| ZJB | 2.96E-03 | 3.31E-03 | 6.27E-03 | 5.44E-04 | 1.09E-03 | 1.63E-03 |
| ZJJ | 1.19E-03 | 1.33E-03 | 2.52E-03 | 2.19E-04 | 4.37E-04 | 6.57E-04 |
| ZZT | 4.53E-03 | 5.07E-03 | 9.61E-03 | 8.34E-04 | 1.66E-03 | 2.50E-03 |
| ZZB | 8.96E-03 | 1.00E-02 | 1.90E-02 | 1.65E-03 | 3.29E-03 | 4.94E-03 |
| ZZS | 2.66E-02 | 2.97E-02 | 5.63E-02 | 4.89E-03 | 9.75E-03 | 1.46E-02 |
| ZZZ | 1.67E-03 | 1.87E-03 | 3.54E-03 | 3.08E-04 | 6.14E-04 | 9.21E-04 |
| SWN | 4.56E-03 | 5.10E-03 | 9.66E-03 | 8.39E-04 | 1.67E-03 | 2.51E-03 |
| BHX | 3.42E-03 | 3.83E-03 | 7.25E-03 | 6.30E-04 | 1.26E-03 | 1.89E-03 |
| STB | 2.74E-03 | 3.07E-03 | 5.81E-03 | 5.04E-04 | 1.01E-03 | 1.51E-03 |
| JYD | 4.20E-03 | 4.71E-03 | 8.91E-03 | 7.74E-04 | 1.54E-03 | 2.32E-03 |

| | | | | | | |
|---|---|---|---|---|---|---|
| GXQZZ | 3.06E-03 | 3.43E-03 | 6.50E-03 | 5.64E-04 | 1.13E-03 | 1.69E-03 |
| DGF | 9.41E-04 | 1.05E-03 | 2.00E-03 | 1.73E-04 | 3.46E-04 | 5.19E-04 |
| JMC | 3.51E-03 | 3.93E-03 | 7.44E-03 | 6.46E-04 | 1.29E-03 | 1.94E-03 |
| ZHG | 3.66E-02 | 4.10E-02 | 7.75E-02 | 6.73E-03 | 1.34E-02 | 2.02E-02 |
| ZSL | 2.62E-02 | 2.94E-02 | 5.56E-02 | 4.83E-03 | 9.63E-03 | 1.45E-02 |
| HZX | 7.76E-03 | 8.69E-03 | 1.65E-02 | 1.43E-03 | 2.85E-03 | 4.28E-03 |
| GZX | 1.21E-03 | 1.36E-03 | 2.57E-03 | 2.23E-04 | 4.45E-04 | 6.67E-04 |
| GZT | 2.99E-03 | 3.35E-03 | 6.34E-03 | 5.50E-04 | 1.10E-03 | 1.65E-03 |
| GZN | 8.17E-03 | 9.16E-03 | 1.73E-02 | 1.51E-03 | 3.00E-03 | 4.51E-03 |
| SZM | 6.37E-03 | 7.13E-03 | 1.35E-02 | 1.17E-03 | 2.34E-03 | 3.51E-03 |
| SZX | 6.59E-03 | 7.38E-03 | 1.40E-02 | 1.21E-03 | 2.42E-03 | 3.63E-03 |
| **maximum** | 3.66E-02 | 4.10E-02 | 7.75E-02 | 6.73E-03 | 1.34E-02 | 2.02E-02 |
| **minimum** | 9.41E-04 | 1.05E-03 | 2.00E-03 | 1.73E-04 | 3.46E-04 | 5.19E-04 |
| **mean** | 6.70E-03 | 7.50E-03 | 1.42E-02 | 1.23E-03 | 2.46E-03 | 3.69E-03 |

**C:The hazard quotient (HQ) of TDCIPP with two pathways in soils**

| **Sampling location** | **children** | | | **adult** | | |
|---|---|---|---|---|---|---|
| | ingestion | Dermal contact | Total HQ | ingestion | Dermal contact | Total HQ |
| CZX | 1.84E-02 | 2.06E-02 | 3.91E-02 | 3.39E-03 | 6.77E-03 | 1.02E-02 |
| FCGS | 8.24E-03 | 9.23E-03 | 1.75E-02 | 1.52E-03 | 3.03E-03 | 4.54E-03 |
| FCGL | 1.21E-02 | 1.35E-02 | 2.56E-02 | 2.23E-03 | 4.44E-03 | 6.67E-03 |
| MMT | 1.39E-02 | 1.56E-02 | 2.95E-02 | 2.56E-03 | 5.12E-03 | 7.68E-03 |
| MMN | 6.90E-03 | 7.73E-03 | 1.46E-02 | 1.27E-03 | 2.54E-03 | 3.81E-03 |
| MMJ | 2.51E-03 | 2.81E-03 | 5.31E-03 | 4.62E-04 | 9.21E-04 | 1.38E-03 |
| MML | 1.28E-02 | 1.43E-02 | 2.71E-02 | 2.35E-03 | 4.69E-03 | 7.04E-03 |
| MMY | 4.17E-03 | 4.67E-03 | 8.84E-03 | 7.68E-04 | 1.53E-03 | 2.30E-03 |
| QZD | 6.63E-03 | 7.43E-03 | 1.41E-02 | 1.22E-03 | 2.44E-03 | 3.66E-03 |
| QZM | 2.00E-02 | 2.24E-02 | 4.25E-02 | 3.69E-03 | 7.36E-03 | 1.10E-02 |
| QZB | 5.92E-03 | 6.63E-03 | 1.26E-02 | 1.09E-03 | 2.17E-03 | 3.27E-03 |
| XMZ | 1.63E-02 | 1.83E-02 | 3.46E-02 | 3.01E-03 | 6.00E-03 | 9.00E-03 |
| XML | 2.03E-02 | 2.28E-02 | 4.31E-02 | 3.74E-03 | 7.47E-03 | 1.12E-02 |
| YJS | 1.34E-03 | 1.50E-03 | 2.83E-03 | 2.46E-04 | 4.91E-04 | 7.37E-04 |
| YJL | 1.91E-02 | 2.14E-02 | 4.05E-02 | 3.51E-03 | 7.01E-03 | 1.05E-02 |
| ZJT | 1.02E-02 | 1.14E-02 | 2.16E-02 | 1.87E-03 | 3.74E-03 | 5.61E-03 |
| ZJB | 1.38E-02 | 1.54E-02 | 2.92E-02 | 2.53E-03 | 5.06E-03 | 7.59E-03 |
| ZJJ | 6.00E-03 | 6.72E-03 | 1.27E-02 | 1.11E-03 | 2.20E-03 | 3.31E-03 |
| ZZT | 1.50E-02 | 1.68E-02 | 3.18E-02 | 2.76E-03 | 5.50E-03 | 8.26E-03 |
| ZZB | 1.15E-02 | 1.28E-02 | 2.43E-02 | 2.11E-03 | 4.21E-03 | 6.32E-03 |
| ZZS | 1.28E-02 | 1.44E-02 | 2.72E-02 | 2.36E-03 | 4.71E-03 | 7.07E-03 |
| ZZZ | 2.85E-03 | 3.19E-03 | 6.03E-03 | 5.24E-04 | 1.05E-03 | 1.57E-03 |
| SWN | 1.65E-02 | 1.84E-02 | 3.49E-02 | 3.03E-03 | 6.05E-03 | 9.08E-03 |
| BHX | 2.42E-03 | 2.71E-03 | 5.14E-03 | 4.46E-04 | 8.90E-04 | 1.34E-03 |
| STB | 9.92E-03 | 1.11E-02 | 2.10E-02 | 1.83E-03 | 3.64E-03 | 5.47E-03 |

| | | | | | | |
|---|---|---|---|---|---|---|
| JYD | 1.59E-02 | 1.79E-02 | 3.38E-02 | 2.94E-03 | 5.86E-03 | 8.79E-03 |
| GXQZZ | 1.38E-02 | 1.54E-02 | 2.92E-02 | 2.54E-03 | 5.07E-03 | 7.61E-03 |
| DGF | 2.46E-03 | 2.76E-03 | 5.22E-03 | 4.54E-04 | 9.05E-04 | 1.36E-03 |
| JMC | 1.01E-02 | 1.13E-02 | 2.14E-02 | 1.86E-03 | 3.71E-03 | 5.57E-03 |
| ZHG | 1.30E-02 | 1.46E-02 | 2.76E-02 | 2.39E-03 | 4.78E-03 | 7.17E-03 |
| ZSL | 1.92E-02 | 2.15E-02 | 4.06E-02 | 3.53E-03 | 7.04E-03 | 1.06E-02 |
| HZX | 1.39E-02 | 1.55E-02 | 2.94E-02 | 2.55E-03 | 5.09E-03 | 7.64E-03 |
| GZX | 8.79E-03 | 9.84E-03 | 1.86E-02 | 1.62E-03 | 3.23E-03 | 4.85E-03 |
| GZT | 9.95E-03 | 1.11E-02 | 2.11E-02 | 1.83E-03 | 3.66E-03 | 5.49E-03 |
| GZN | 1.62E-02 | 1.81E-02 | 3.42E-02 | 2.97E-03 | 5.93E-03 | 8.91E-03 |
| SZM | 1.64E-02 | 1.84E-02 | 3.49E-02 | 3.03E-03 | 6.04E-03 | 9.07E-03 |
| SZX | 1.66E-02 | 1.86E-02 | 3.52E-02 | 3.06E-03 | 6.10E-03 | 9.15E-03 |
| **maximum** | 2.03E-02 | 2.28E-02 | 4.31E-02 | 3.74E-03 | 7.47E-03 | 1.12E-02 |
| **minimum** | 1.34E-03 | 1.50E-03 | 2.83E-03 | 2.46E-04 | 4.91E-04 | 7.37E-04 |
| **mean** | 1.15E-02 | 1.29E-02 | 2.44E-02 | 2.12E-03 | 4.23E-03 | 6.35E-03 |

**D:The hazard quotient (HQ) of TPHP with two pathways in soils**

| **Sampling location** | **children** | | | **adult** | | |
|---|---|---|---|---|---|---|
| | ingestion | Dermal contact | Total HQ | ingestion | Dermal contact | Total HQ |
| CZX | 2.21E-04 | 2.47E-04 | 4. 68E-04 | 4.06E-05 | 8.10253E-05 | 1. 22E-04 |
| FCGS | 1.72E-04 | 1.92E-04 | 3. 64E-04 | 3.16E-05 | 6.30409E-05 | 9. 46E-05 |
| FCGL | 2.28E-04 | 2.55E-04 | 4. 83E-04 | 4.19E-05 | 8.36426E-05 | 1. 26E-04 |
| MMT | 1.89E-04 | 2.12E-04 | 4. 01E-04 | 3.48E-05 | 6.95013E-05 | 1. 04E-04 |
| MMN | 3.31E-04 | 3.71E-04 | 7. 02E-04 | 6.10E-05 | 0.000121708 | 1. 83E-04 |
| MMJ | 2.12E-04 | 2.37E-04 | 4. 48E-04 | 3.89E-05 | 7.7693E-05 | 1. 17E-04 |
| MML | 3.89E-04 | 4.36E-04 | 8. 25E-04 | 7.16E-05 | 0.000142902 | 2. 15E-04 |
| MMY | 0 | 0 | 0 | 0 | 0 | 0 |
| QZD | 1.80E-04 | 2.02E-04 | 3. 83E-04 | 3.32E-05 | 6.62961E-05 | 9. 95E-05 |
| QZM | 1.71E-04 | 1.91E-04 | 3. 62E-04 | 3.14E-05 | 6.27168E-05 | 9. 42E-05 |
| QZB | 2.31E-04 | 2.59E-04 | 4. 90E-04 | 4.25E-05 | 8.48566E-05 | 1. 27E-04 |
| XMZ | 1.62E-04 | 1.82E-04 | 3. 44E-04 | 2.99E-05 | 5.96104E-05 | 8. 95E-05 |
| XML | 1.77E-04 | 1.98E-04 | 3. 75E-04 | 3.26E-05 | 6.49888E-05 | 9. 76E-05 |
| YJS | 1.79E-04 | 2.00E-04 | 3. 79E-04 | 3.29E-05 | 6.56536E-05 | 9. 86E-05 |
| YJL | 1.63E-04 | 1.82E-04 | 3. 45E-04 | 3.00E-05 | 5.97811E-05 | 8. 97E-05 |
| ZJT | 1.86E-04 | 2.09E-04 | 3. 95E-04 | 3.43E-05 | 6.8507E-05 | 1. 03E-04 |
| ZJB | 2.27E-04 | 2.55E-04 | 4. 82E-04 | 4.19E-05 | 8.35148E-05 | 1. 25E-04 |
| ZJJ | 2.44E-04 | 2.73E-04 | 5. 17E-04 | 4.49E-05 | 8.94949E-05 | 1. 34E-04 |
| ZZT | 1.96E-04 | 2.19E-04 | 4. 15E-04 | 3.60E-05 | 7.18283E-05 | 1. 08E-04 |
| ZZB | 1.63E-04 | 1.82E-04 | 3. 45E-04 | 3.00E-05 | 5.97644E-05 | 8. 97E-05 |
| ZZS | 2.00E-04 | 2.24E-04 | 4. 24E-04 | 3.68E-05 | 7.34018E-05 | 1. 10E-04 |
| ZZZ | 9.35E-05 | 1.05E-04 | 1. 98E-04 | 1.72E-05 | 3.43468E-05 | 5. 16E-05 |
| SWN | 1.88E-04 | 2.10E-04 | 3. 98E-04 | 3.45E-05 | 6.88822E-05 | 1. 03E-04 |
| BHX | 1.57E-04 | 1.76E-04 | 3. 33E-04 | 2.89E-05 | 5.77546E-05 | 8. 67E-05 |

| | | | | | | |
|---|---|---|---|---|---|---|
| STB | 1.94E-04 | 2.17E-04 | 4.11E-04 | 3.57E-05 | 7.12623E-05 | 1.07E-04 |
| JYD | 1.46E-04 | 1.63E-04 | 3.09E-04 | 2.68E-05 | 5.3513E-05 | 8.03E-05 |
| GXQZZ | 1.57E-04 | 1.76E-04 | 3.34E-04 | 2.90E-05 | 5.78484E-05 | 8.68E-05 |
| DGF | 1.55E-04 | 1.73E-04 | 3.28E-04 | 2.85E-05 | 5.6786E-05 | 8.53E-05 |
| JMC | 3.35E-04 | 3.75E-04 | 7.10E-04 | 6.16E-05 | 0.000122952 | 1.85E-04 |
| ZHG | 1.06E-04 | 1.18E-04 | 2.24E-04 | 1.94E-05 | 3.87592E-05 | 5.82E-05 |
| ZSL | 1.86E-04 | 2.09E-04 | 3.95E-04 | 3.43E-05 | 6.8433E-05 | 1.03E-04 |
| HZX | 1.10E-04 | 1.23E-04 | 2.33E-04 | 2.02E-05 | 4.0325E-05 | 6.05E-05 |
| GZX | 1.55E-04 | 1.74E-04 | 3.29E-04 | 2.86E-05 | 5.69932E-05 | 8.56E-05 |
| GZT | 1.36E-04 | 1.53E-04 | 2.89E-04 | 2.51E-05 | 5.00775E-05 | 7.52E-05 |
| GZN | 1.74E-04 | 1.95E-04 | 3.69E-04 | 3.20E-05 | 6.39173E-05 | 9.60E-05 |
| SZM | 3.25E-04 | 3.64E-04 | 6.89E-04 | 5.99E-05 | 0.000119443 | 1.79E-04 |
| SZX | 1.16E-04 | 1.30E-04 | 2.46E-04 | 2.14E-05 | 4.26535E-05 | 6.40E-05 |
| **maximum** | 3.89E-04 | 4.36E-04 | 8.25E-04 | 7.16E-05 | 1.43E-04 | 2.15E-04 |
| **minimum** | 0 | 0 | 0 | 0 | 0 | 0 |
| **mean** | 1.88E-04 | 2.10E-04 | 3.98E-04 | 3.46E-05 | 6.90E-05 | 1.04E-04 |

**Table S8 The carcinogenic risk values (CR) for different OPEs with two pathways in soils.**

**A:The carcinogenic risk values (CR) of TCEP with two pathways in soils**

| **Sampling location** | **children** | | | **adult** | | |
|---|---|---|---|---|---|---|
| | ingestion | Dermal contact | Total CR | ingestion | Dermal contact | Total CR |
| CZX | 2.61E-07 | 2.93E-07 | 5.54E-07 | 1.93E-07 | 3.84E-07 | 5.77E-07 |
| FCGS | 3.72E-08 | 4.17E-08 | 7.89E-08 | 2.74E-08 | 5.47E-08 | 8.21E-08 |
| FCGL | 4.64E-08 | 5.20E-08 | 9.84E-08 | 3.42E-08 | 6.82E-08 | 1.02E-07 |
| MMT | 4.92E-08 | 5.52E-08 | 1.04E-07 | 3.63E-08 | 7.24E-08 | 1.09E-07 |
| MMN | 3.61E-08 | 4.04E-08 | 7.65E-08 | 2.66E-08 | 5.31E-08 | 7.96E-08 |
| MMJ | 2.83E-08 | 3.17E-08 | 5.99E-08 | 2.08E-08 | 4.15E-08 | 6.23E-08 |
| MML | 4.75E-08 | 5.31E-08 | 1.01E-07 | 3.49E-08 | 6.97E-08 | 1.05E-07 |
| MMY | 3.18E-08 | 3.56E-08 | 6.74E-08 | 2.34E-08 | 4.67E-08 | 7.02E-08 |
| QZD | 4.62E-08 | 5.17E-08 | 9.79E-08 | 3.40E-08 | 6.78E-08 | 1.02E-07 |
| QZM | 8.65E-08 | 9.68E-08 | 1.83E-07 | 6.37E-08 | 1.27E-07 | 1.91E-07 |
| QZB | 6.35E-08 | 7.11E-08 | 1.35E-07 | 4.68E-08 | 9.33E-08 | 1.40E-07 |
| XMZ | 6.80E-08 | 7.62E-08 | 1.44E-07 | 5.01E-08 | 9.99E-08 | 1.50E-07 |
| XML | 5.70E-08 | 6.38E-08 | 1.21E-07 | 4.20E-08 | 8.37E-08 | 1.26E-07 |
| YJS | 2.84E-08 | 3.18E-08 | 6.02E-08 | 2.09E-08 | 4.17E-08 | 6.26E-08 |
| YJL | 7.93E-08 | 8.88E-08 | 1.68E-07 | 5.84E-08 | 1.17E-07 | 1.75E-07 |
| ZJT | 3.71E-08 | 4.16E-08 | 7.87E-08 | 2.73E-08 | 5.45E-08 | 8.19E-08 |
| ZJB | 3.55E-08 | 3.97E-08 | 7.52E-08 | 2.61E-08 | 5.21E-08 | 7.82E-08 |
| ZJJ | 1.43E-08 | 1.60E-08 | 3.03E-08 | 1.05E-08 | 2.10E-08 | 3.15E-08 |
| ZZT | 5.44E-08 | 6.09E-08 | 1.15E-07 | 4.00E-08 | 7.99E-08 | 1.20E-07 |
| ZZB | 1.08E-07 | 1.20E-07 | 2.28E-07 | 7.92E-08 | 1.58E-07 | 2.37E-07 |

| | | | | | | |
|---|---|---|---|---|---|---|
| ZZS | 3.19E-07 | 3.57E-07 | 6.75E-07 | 2.35E-07 | 4.68E-07 | 7.03E-07 |
| ZZZ | 2.00E-08 | 2.24E-08 | 4.25E-08 | 1.48E-08 | 2.95E-08 | 4.42E-08 |
| SWN | 5.47E-08 | 6.13E-08 | 1.16E-07 | 4.03E-08 | 8.04E-08 | 1.21E-07 |
| BHX | 4.11E-08 | 4.60E-08 | 8.70E-08 | 3.02E-08 | 6.03E-08 | 9.06E-08 |
| STB | 3.29E-08 | 3.68E-08 | 6.97E-08 | 2.42E-08 | 4.83E-08 | 7.25E-08 |
| JYD | 5.04E-08 | 5.65E-08 | 1.07E-07 | 3.71E-08 | 7.41E-08 | 1.11E-07 |
| GXQZZ | 3.68E-08 | 4.12E-08 | 7.80E-08 | 2.71E-08 | 5.40E-08 | 8.11E-08 |
| DGF | 1.13E-08 | 1.27E-08 | 2.39E-08 | 8.32E-09 | 1.66E-08 | 2.49E-08 |
| JMC | 4.21E-08 | 4.72E-08 | 8.93E-08 | 3.10E-08 | 6.19E-08 | 9.29E-08 |
| ZHG | 4.39E-07 | 4.91E-07 | 9.30E-07 | 3.23E-07 | 6.45E-07 | 9.68E-07 |
| ZSL | 3.15E-07 | 3.52E-07 | 6.67E-07 | 2.32E-07 | 4.62E-07 | 6.94E-07 |
| HZX | 9.31E-08 | 1.04E-07 | 1.97E-07 | 6.86E-08 | 1.37E-07 | 2.05E-07 |
| GZX | 1.45E-08 | 1.63E-08 | 3.08E-08 | 1.07E-08 | 2.13E-08 | 3.20E-08 |
| GZT | 3.59E-08 | 4.02E-08 | 7.60E-08 | 2.64E-08 | 5.27E-08 | 7.91E-08 |
| GZN | 9.81E-08 | 1.10E-07 | 2.08E-07 | 7.22E-08 | 1.44E-07 | 2.16E-07 |
| SZM | 7.64E-08 | 8.56E-08 | 1.62E-07 | 5.63E-08 | 1.12E-07 | 1.69E-07 |
| SZX | 7.91E-08 | 8.85E-08 | 1.68E-07 | 5.82E-08 | 1.16E-07 | 1.74E-07 |
| **maximum** | 4.39E-07 | 4.91E-07 | 9.30E-07 | 3.23E-07 | 6.45E-07 | 9.68E-07 |
| **minimum** | 1.13E-08 | 1.27E-08 | 2.39E-08 | 8.32E-09 | 1.66E-08 | 2.49E-08 |
| **mean** | 8.04E-08 | 9.00E-08 | 1.70E-07 | 5.92E-08 | 1.18E-07 | 1.77E-07 |

**B:The carcinogenic risk values (CR) of TDCIPP with two pathways in soils**

| **Sampling location** | **children** | | | **adult** | | |
|---|---|---|---|---|---|---|
| | ingestion | Dermal contact | Total CR | ingestion | Dermal contact | Total CR |
| CZX | 9.79E-07 | 4.54E-07 | 1.43E-06 | 7.21E-07 | 1.44E-06 | 2.16E-06 |
| FCGS | 4.38E-07 | 6.46E-08 | 5.02E-07 | 3.22E-07 | 6.43E-07 | 9.66E-07 |
| FCGL | 6.43E-07 | 8.06E-08 | 7.23E-07 | 4.73E-07 | 9.44E-07 | 1.42E-06 |
| MMT | 7.40E-07 | 8.55E-08 | 8.26E-07 | 5.45E-07 | 1.09E-06 | 1.63E-06 |
| MMN | 3.67E-07 | 6.27E-08 | 4.30E-07 | 2.70E-07 | 5.39E-07 | 8.09E-07 |
| MMJ | 1.33E-07 | 4.91E-08 | 1.82E-07 | 9.81E-08 | 1.96E-07 | 2.94E-07 |
| MML | 6.78E-07 | 8.24E-08 | 7.61E-07 | 4.99E-07 | 9.96E-07 | 1.50E-06 |
| MMY | 2.22E-07 | 5.52E-08 | 2.77E-07 | 1.63E-07 | 3.26E-07 | 4.89E-07 |
| QZD | 3.53E-07 | 8.02E-08 | 4.33E-07 | 2.60E-07 | 5.18E-07 | 7.78E-07 |
| QZM | 1.06E-06 | 1.50E-07 | 1.21E-06 | 7.84E-07 | 1.56E-06 | 2.35E-06 |
| QZB | 3.15E-07 | 1.10E-07 | 4.25E-07 | 2.32E-07 | 4.62E-07 | 6.94E-07 |
| XMZ | 8.68E-07 | 1.18E-07 | 9.86E-07 | 6.39E-07 | 1.27E-06 | 1.91E-06 |
| XML | 1.08E-06 | 9.89E-08 | 1.18E-06 | 7.95E-07 | 1.59E-06 | 2.38E-06 |
| YJS | 7.10E-08 | 4.93E-08 | 1.20E-07 | 5.23E-08 | 1.04E-07 | 1.57E-07 |
| YJL | 1.01E-06 | 1.38E-07 | 1.15E-06 | 7.47E-07 | 1.49E-06 | 2.24E-06 |
| ZJT | 5.40E-07 | 6.44E-08 | 6.05E-07 | 3.98E-07 | 7.94E-07 | 1.19E-06 |
| ZJB | 7.32E-07 | 6.16E-08 | 7.93E-07 | 5.39E-07 | 1.08E-06 | 1.61E-06 |

| | | | | | | |
|---|---|---|---|---|---|---|
| ZJJ | 3.19E-07 | 2.48E-08 | 3.44E-07 | 2.35E-07 | 4.69E-07 | 7.04E-07 |
| ZZT | 7.96E-07 | 9.44E-08 | 8.91E-07 | 5.86E-07 | 1.17E-06 | 1.76E-06 |
| ZZB | 6.09E-07 | 1.87E-07 | 7.96E-07 | 4.48E-07 | 8.95E-07 | 1.34E-06 |
| ZZS | 6.82E-07 | 5.53E-07 | 1.23E-06 | 5.02E-07 | 1.00E-06 | 1.50E-06 |
| ZZZ | 1.51E-07 | 3.48E-08 | 1.86E-07 | 1.11E-07 | 2.22E-07 | 3.34E-07 |
| SWN | 8.75E-07 | 9.49E-08 | 9.70E-07 | 6.44E-07 | 1.29E-06 | 1.93E-06 |
| BHX | 1.29E-07 | 7.13E-08 | 2.00E-07 | 9.48E-08 | 1.89E-07 | 2.84E-07 |
| STB | 5.27E-07 | 5.71E-08 | 5.84E-07 | 3.88E-07 | 7.75E-07 | 1.16E-06 |
| JYD | 8.47E-07 | 8.75E-08 | 9.35E-07 | 6.24E-07 | 1.24E-06 | 1.87E-06 |
| GXQZZ | 7.33E-07 | 6.38E-08 | 7.97E-07 | 5.40E-07 | 1.08E-06 | 1.62E-06 |
| DGF | 1.31E-07 | 1.96E-08 | 1.51E-07 | 9.64E-08 | 1.92E-07 | 2.89E-07 |
| JMC | 5.37E-07 | 7.31E-08 | 6.10E-07 | 3.95E-07 | 7.89E-07 | 1.18E-06 |
| ZHG | 6.91E-07 | 7.62E-07 | 1.45E-06 | 5.09E-07 | 1.02E-06 | 1.52E-06 |
| ZSL | 1.02E-06 | 5.46E-07 | 1.56E-06 | 7.50E-07 | 1.50E-06 | 2.25E-06 |
| HZX | 7.36E-07 | 1.62E-07 | 8.98E-07 | 5.42E-07 | 1.08E-06 | 1.62E-06 |
| GZX | 4.67E-07 | 2.52E-08 | 4.92E-07 | 3.44E-07 | 6.86E-07 | 1.03E-06 |
| GZT | 5.29E-07 | 6.23E-08 | 5.91E-07 | 3.89E-07 | 7.77E-07 | 1.17E-06 |
| GZN | 8.58E-07 | 1.70E-07 | 1.03E-06 | 6.32E-07 | 1.26E-06 | 1.89E-06 |
| SZM | 8.74E-07 | 1.33E-07 | 1.01E-06 | 6.43E-07 | 1.28E-06 | 1.93E-06 |
| SZX | 8.82E-07 | 1.37E-07 | 1.02E-06 | 6.50E-07 | 1.30E-06 | 1.95E-06 |
| **maximum** | 1.08E-06 | 7.62E-07 | 1.56E-06 | 7.95E-07 | 1.59E-06 | 2.38E-06 |
| **minimum** | 7.10E-08 | 1.96E-08 | 1.20E-07 | 5.23E-08 | 1.04E-07 | 1.57E-07 |
| **mean** | 6.12E-07 | 1.40E-07 | 7.51E-07 | 4.50E-07 | 8.99E-07 | 1.35E-06 |

**C: The carcinogenic risk values (CR) of ΣOPE with two pathways in soils**

| **Sampling location** | **children** | | | **adult** | | |
|---|---|---|---|---|---|---|
| | ingestion | Dermal contact | Total CR | ingestion | Dermal contact | Total CR |
| CZX | 1.24E-06 | 7.47E-07 | 1.99E-06 | 9.14E-07 | 1.82E-06 | 2.74E-06 |
| FCGS | 4.75E-07 | 1.06E-07 | 5.81E-07 | 3.50E-07 | 6.98E-07 | 1.05E-06 |
| FCGL | 6.89E-07 | 1.33E-07 | 8.22E-07 | 5.08E-07 | 1.01E-06 | 1.52E-06 |
| MMT | 7.90E-07 | 1.41E-07 | 9.30E-07 | 5.82E-07 | 1.16E-06 | 1.74E-06 |
| MMN | 4.03E-07 | 1.03E-07 | 5.06E-07 | 2.97E-07 | 5.92E-07 | 8.89E-07 |
| MMJ | 1.61E-07 | 8.07E-08 | 2.42E-07 | 1.19E-07 | 2.37E-07 | 3.56E-07 |
| MML | 7.26E-07 | 1.36E-07 | 8.61E-07 | 5.34E-07 | 1.07E-06 | 1.60E-06 |
| MMY | 2.54E-07 | 9.08E-08 | 3.44E-07 | 1.87E-07 | 3.73E-07 | 5.59E-07 |
| QZD | 3.99E-07 | 1.32E-07 | 5.31E-07 | 2.94E-07 | 5.86E-07 | 8.80E-07 |
| QZM | 1.15E-06 | 2.47E-07 | 1.40E-06 | 8.48E-07 | 1.69E-06 | 2.54E-06 |
| QZB | 3.78E-07 | 1.81E-07 | 5.60E-07 | 2.79E-07 | 5.56E-07 | 8.34E-07 |
| XMZ | 9.36E-07 | 1.94E-07 | 1.13E-06 | 6.89E-07 | 1.37E-06 | 2.06E-06 |
| XML | 1.14E-06 | 1.63E-07 | 1.30E-06 | 8.37E-07 | 1.67E-06 | 2.51E-06 |
| YJS | 9.94E-08 | 8.11E-08 | 1.80E-07 | 7.32E-08 | 1.46E-07 | 2.19E-07 |
| YJL | 1.09E-06 | 2.26E-07 | 1.32E-06 | 8.06E-07 | 1.61E-06 | 2.41E-06 |

| | | | | | | |
|---|---|---|---|---|---|---|
| ZJT | 5.78E-07 | 1.06E-07 | 6.84E-07 | 4.25E-07 | 8.49E-07 | 1.27E-06 |
| ZJB | 7.67E-07 | 1.01E-07 | 8.68E-07 | 5.65E-07 | 1.13E-06 | 1.69E-06 |
| ZJJ | 3.33E-07 | 4.08E-08 | 3.74E-07 | 2.45E-07 | 4.90E-07 | 7.35E-07 |
| ZZT | 8.51E-07 | 1.55E-07 | 1.01E-06 | 6.26E-07 | 1.25E-06 | 1.88E-06 |
| ZZB | 7.16E-07 | 3.07E-07 | 1.02E-06 | 5.28E-07 | 1.05E-06 | 1.58E-06 |
| ZZS | 1.00E-06 | 9.10E-07 | 1.91E-06 | 7.37E-07 | 1.47E-06 | 2.21E-06 |
| ZZZ | 1.71E-07 | 5.72E-08 | 2.29E-07 | 1.26E-07 | 2.52E-07 | 3.78E-07 |
| SWN | 9.30E-07 | 1.56E-07 | 1.09E-06 | 6.85E-07 | 1.37E-06 | 2.05E-06 |
| BHX | 1.70E-07 | 1.17E-07 | 2.87E-07 | 1.25E-07 | 2.49E-07 | 3.75E-07 |
| STB | 5.60E-07 | 9.39E-08 | 6.54E-07 | 4.12E-07 | 8.23E-07 | 1.24E-06 |
| JYD | 8.98E-07 | 1.44E-07 | 1.04E-06 | 6.61E-07 | 1.32E-06 | 1.98E-06 |
| GXQZZ | 7.70E-07 | 1.05E-07 | 8.75E-07 | 5.67E-07 | 1.13E-06 | 1.70E-06 |
| DGF | 1.42E-07 | 3.23E-08 | 1.74E-07 | 1.05E-07 | 2.09E-07 | 3.14E-07 |
| JMC | 5.79E-07 | 1.20E-07 | 6.99E-07 | 4.26E-07 | 8.51E-07 | 1.28E-06 |
| ZHG | 1.13E-06 | 1.25E-06 | 2.38E-06 | 8.32E-07 | 1.66E-06 | 2.49E-06 |
| ZSL | 1.33E-06 | 8.99E-07 | 2.23E-06 | 9.82E-07 | 1.96E-06 | 2.94E-06 |
| HZX | 8.29E-07 | 2.66E-07 | 1.10E-06 | 6.11E-07 | 1.22E-06 | 1.83E-06 |
| GZX | 4.82E-07 | 4.15E-08 | 5.23E-07 | 3.55E-07 | 7.08E-07 | 1.06E-06 |
| GZT | 5.65E-07 | 1.02E-07 | 6.67E-07 | 4.16E-07 | 8.30E-07 | 1.25E-06 |
| GZN | 9.56E-07 | 2.80E-07 | 1.24E-06 | 7.04E-07 | 1.41E-06 | 2.11E-06 |
| SZM | 9.50E-07 | 2.18E-07 | 1.17E-06 | 7.00E-07 | 1.40E-06 | 2.10E-06 |
| SZX | 9.61E-07 | 2.26E-07 | 1.19E-06 | 7.08E-07 | 1.41E-06 | 2.12E-06 |
| **maximum** | 1.52E-06 | 1.25E-06 | 2.38E-06 | 1.12E-06 | 2.23E-06 | 2.94E-06 |
| **minimum** | 8.23E-08 | 3.23E-08 | 1.74E-07 | 6.06E-08 | 1.21E-07 | 2.19E-07 |
| **mean** | 6.92E-07 | 2.30E-07 | 9.22E-07 | 5.10E-07 | 1.02E-06 | 1.53E-06 |

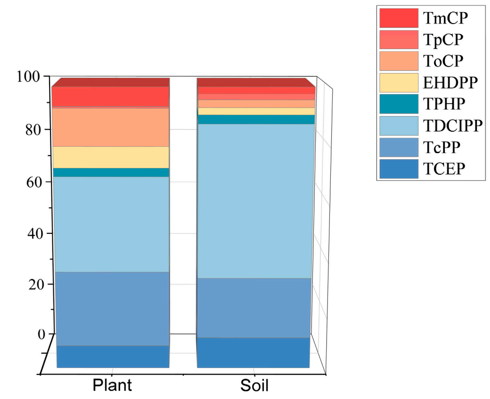


**Figure S1. Contribution degree of each OPE monomer in soil and plants**

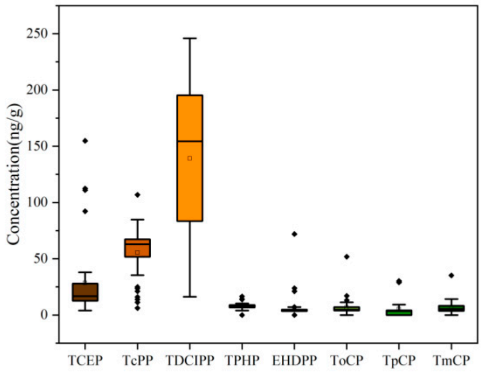


**Figure S2 Concentration of each OPE in the soil**

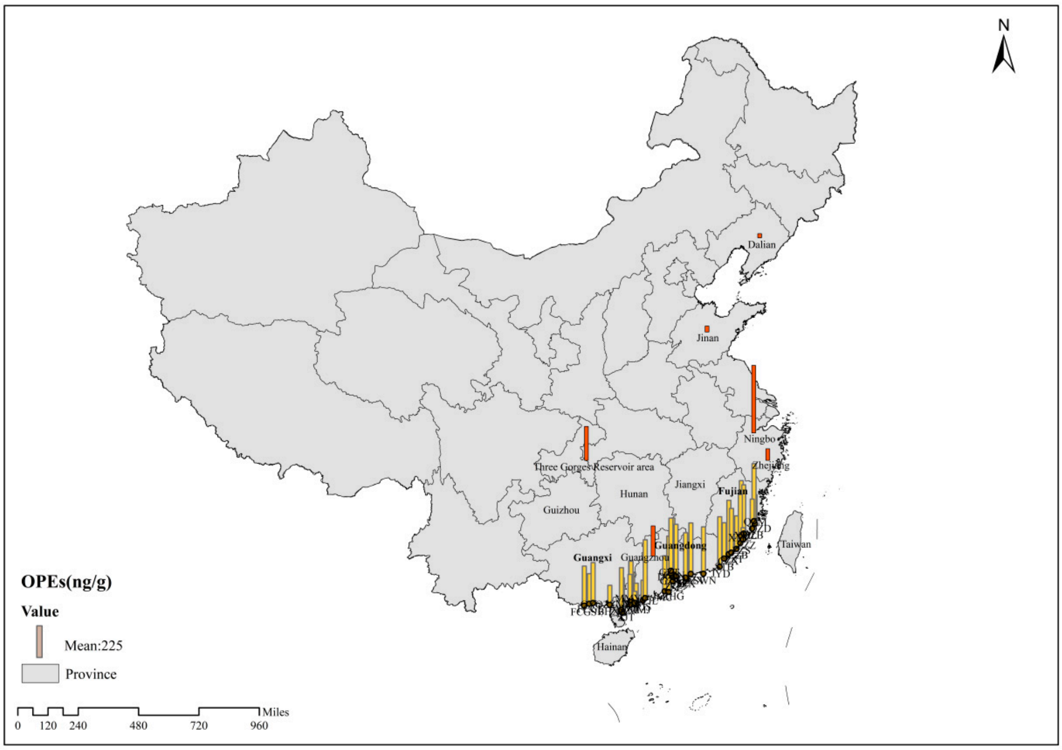


**Figure S3 Concentration and geographical distribution of OPEs in soils in coastal areas of South China and other studied regions. The concentration of OPEs in Other studied regions (red column); The concentration of OPEs in this study (yellow column).**

.

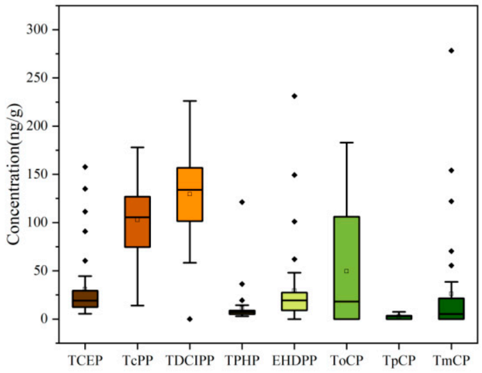


**Figure S4 Concentration of each OPE in the plant**

Reference:
[1] Fu, L.F.; Du, B.B.; Wang, F.; Lam, J.C. W.; Zeng, L.X.; Zeng, E.Y. Organophosphate tri-esters and di-ester degradation products in municipal sludge from wastewater treatment plants in China: Spatial patterns and ecological implications. *Environ. Sci. Technol*, 2017, 51, 13614-13623.
[2]European Commission. Identification and Evaluation of Data On flame Retardants in Consumer Products. **2017**. Contract Number 17.020200/09/549040. Available online: https://www.europeanfiresafetyalliance.org/publications/identification-and-evaluation-of-data-on-flame-retardants-in-consumer-products/ (accessed on 12 January 2024).
[3] USEPA Mid Atlantic Risk Assessment, Regional Screening Levels (RSLs) - Generic Tables. **2023**. http://www.epa.gov/region9/superfund/prg (accessed on 16 January 2024).
[4] Ding, J.J.; Shen, X.L.; Liu, W.P.; Covaci, A.; Yang, F.X. Occurrence and risk assessment of organophosphate esters in drinking water from Eastern China. *Sci Total Environ*, **2015**, 538, 959-965.
[5] Ali, N.; Dirtu, A.C. .; Eede, N.V.D.; Goosey, E.; Harrad, S.; Neels, H.; Mannetje, A.; Coakley, J.; Douwes, J.; Covaci, A. Occurrence of alternative flame retardants in indoor dust from New Zealand: Indoor sources and human exposure assessment. *Chemosphere*, **2012**, 88, 1276-1282.
[6] USEPA Exposure Factors Handbook, final ed. US Environmental Protection Agency. **2011**. Washington, DC [EPA/600/R-09/052F].
[7] USDoE. The Risk Assessment Information System (RAIS). U.S. Department of Energy’s Oak Ridge Operations Office (ORO). **2011**.
[8] MEPC, Ministry of Environmental Protection of the People's Republic of China Exposure Factors Handbook of Chinese Population. China Environmental Science Press, Beijing, China. **2013**.
[9] USEPA (US Environmental Protection Agency) Supplemental guidance for developing soil screening levels for superfund sites. OSWER9355.4-24. Office of Solid Waste and Emergency Response. US Environmental Protection Agency. Washington, DC. **2001**.
[10]59.Liu, C.Y.; Wang, L.M.; Gao, X.X.; Huang, Z.J.; Zhang, X.; Zhao, Z.P.; Li, C.; Zhang, M. Intake of vegetables and fruit among adults in China in 2018. *Chin J Prev Contr Chron Dis*, **2022**, 30, 561-566.
[11]60.L, L.; Ouyang, Y.F.; Wang, H.J.; H, F.F.; Wang, Y.; Zhang, J.G.; Sui, C.; Du, W.W.; Jia, X.F.; Jiang, H.R.; Wang, Z.H.; Zhang, B. Status of fruit and vegetable intake among children and adolescents in 15 provinces of China. *Chinese Journal of Health Education*, **2020**, 36, 3-7.
